# N6-methyladenosine-modified oncofetal lncRNA MIR4435-2HG contributed to stemness features of hepatocellular carcinoma cells by regulating rRNA 2′-O methylation

**DOI:** 10.1186/s11658-023-00493-2

**Published:** 2023-10-27

**Authors:** Yiqing Zhu, Bang Xiao, Meng Liu, Meiting Chen, Ningqi Xia, Haiyan Guo, Jinfeng Huang, Zhiyong Liu, Fang Wang

**Affiliations:** 1 Department of Medical Genetics, Naval Medical University, Shanghai, 200433 China; 2 Department of Assisted Reproduction, Shanghai Ninth People’s Hospital, Shanghai Jiaotong University School of Medicine, Center for Specialty Strategy Research of Shanghai Jiao Tong University China Hospital Development Institute, Shanghai, 200011 China; 3 Department of Urology, Shanghai Changhai Hospital, Naval Medical University, Shanghai, 200433 China

**Keywords:** Oncofetal lncRNA, Hepatocellular carcinoma, Stemness maintenance, rRNA 2′-O-methylation, N6-methyladenosine

## Abstract

**Background:**

The unique expression pattern endows oncofetal genes with great value in cancer diagnosis and treatment. However, only a few oncofetal genes are available for clinical use and the underlying mechanisms that drives the fetal-like reprogramming of cancer cells remain largely unknown.

**Methods:**

Microarray assays and bioinformatic analyses were employed to screen for potential oncofetal long non-coding RNAs (lncRNAs) in hepatocellular carcinoma (HCC). The expression levels of MIR4435-2HG, NOP58 ribonucleoprotein (NOP58), insulin like growth factor 2 mRNA binding protein 1 (IGF2BP1) and stem markers were detected by quantitative polymerase chain reaction. The 2′-O-methylation (2′-O-Me) status of rRNA were detected through reverse transcription at low dNTP concentrations followed by PCR. The regulation of MIR4435-2HG by IGF2BP1 was explored by RNA immunoprecipitation (RIP), methylated RIP (MeRIP) and dual-luciferase assays. The interaction between MIR4435-2HG and NOP58 was investigated by RNA Pulldown, RIP and protein stability assays. In vitro and in vivo function assays were performed to detect the roles of MIR4435-2HG/NOP58 in HCC.

**Results:**

MIR4435-2HG was an oncofetal lncRNA associated with poor prognosis in HCC. Functional experiments showed that overexpression of MIR4435-2HG remarkably enhanced the stem-cell properties of HCC cells, promoting tumorigenesis in vitro and in vivo. Mechanically, MIR4435-2HG directly bound NOP58 and IGF2BP1. IGF2BP1 upregulated MIR4435-2HG expression in HCC through N6-methyladenosine (m6A) modification. Moreover, MIR4435-2HG protected NOP58 from degradation, which raised rRNA 2’-O-Me levels and promoted internal ribosome entry site (IRES)-dependent translation of oncogenes.

**Conclusions:**

This study identified an oncofetal lncRNA MIR4435-2HG, characterized the role of MIR4435-2HG/NOP58 in stemness maintenance and proliferation of HCC cells, and confirmed m6A as a ‘driver’ that reactivated MR4435-2HG expression in HCC.

**Supplementary Information:**

The online version contains supplementary material available at 10.1186/s11658-023-00493-2.

## Background

Hepatocellular carcinoma (HCC), a major health problem worldwide, ranks as the third leading cause of death from cancer globally [1]. As great advances have been achieved in HCC treatments recently, early-stage HCC patients are eligible for curative therapies, such as surgical resection and liver transplantation [2]. However, few therapies show efficiency for HCC patients diagnosed at advance stages and the current treatment is associated with a high recurrence rate. These emphasize the urgent need for reliable biomarkers and effective intervention strategies and highlight the importance of a better understanding of the precise molecular events involved in the initiation and progression of HCC.

Accumulating evidence has suggested that embryonic development and tumorigenesis share many similarities in both biological behavior and molecular features [3, 4]. The typical features of embryonic stem cells, such as sustaining self-renewal and high cell plasticity, are pivotal for tumor initiation, malignancy and even therapeutic resistance [5]. Meanwhile, a number of genes and signaling pathways involved in critical stages of embryonic development are aberrantly reactivated and play important roles in tumor initiation and progression. These intersections between embryonic development and tumorigenesis shed a new light on our understanding of cancer biology and treatment. In HCC, several oncofetal genes, such as AFP, GPC3 and SALL4, have been identified [6–8]. However, well characterized oncofetal genes for clinical use are still limited, the specific role of oncofetal genes in linking phenotypic characteristics of tumorigenesis with early developmental programs remains elusive, and the ‘drivers’ beneath the reactivation of oncofetal genes are largely unknown.

Long non-coding RNAs (lncRNAs) are an important subtype of non-coding transcripts that exceed 200 nucleotides in length [9]. LncRNAs are implicated in various biological processes and dysregulation of lncRNAs is associated with diverse human diseases, including HCC [10, 11]. Emerging evidence has shown that several lncRNAs mimic the expression pattern of oncofetal genes and contribute to the initiation and progression of HCC [12]. To screen for potential oncofetal lncRNAs, our previous study performed transcriptome-wide sequencing of mouse fetal liver, adult liver and liver tumor tissues [13, 14]. However, as lncRNAs are less conserved between species, we only confirmed the oncogenic role of lncRNA-PVT1 in human HCC [14]. These suggest that more efforts should be employed to identify clinical available oncofetal lncRNAs, which might provide new opportunities for HCC treatment and a better understanding of the oncofetal reprogramming of HCC cells.

In this manuscript, we identified an oncofetal lncRNA, MIR4435-2HG, which promoted HCC cell growth and strengthened the stemness features of HCC cells in vitro and in vivo. Mechanistically, MIR4435-2HG directly interacted with NOP58 ribonucleoprotein (NOP58) and insulin like growth factor 2 mRNA binding protein 1 (IGF2BP1). IGF2BP1 unregulated MIR4435-2HG expression in HCC cells through N6-methyladenosine (m6A) modification. Meanwhile, MIR4435-2HG protected NOP58 from degradation. Further study revealed that NOP58 contributed to the initiation and progression of HCC through increasing the rRNA 2′-O-methylation (2′-O-Me) and activating internal ribosome entry site (IRES)-dependent translation initiation of oncogenes. Collectively, MIR4435-2HG has shown great potential as a prognosis biomarker and therapeutic target for HCC.

## Materials and methods

### Clinical samples

A total of 88 paired tumor and nontumor tissues were collected from well informed HCC patients in the Eastern Hepatobiliary Surgery Hospital (Naval Medical University, Shanghai, China). Fetal liver tissues were collected from well informed patients who receipt pregnancy termination in the Changzheng Hospital (Naval Medical University, Shanghai, China). Ethical consent was granted from the Committee on Ethics of Biomedicine, Naval Medical University.

### LncRNA microarray analysis

Biological triplicates of both fetal liver and adult liver tissues were used for lncRNA profiling by microarray analysis. In brief, total RNA was extracted, labeled cRNA was prepared, and RNA hybridization was conducted using Human LncRNA 3.0 (8 × 60k, Arraystar, USA) by Aksomics (Shanghai, China). Array images were acquired by Agilent Feature Extraction software and further analyzed using GeneSpring GX v11.5.1 software package (Agilent Technologies, USA).

### Cell culture

Human hepatoma cell line Huh7 (TCHu182) and HepG2 (TCHu 72) and human embryonic kidney cell line 293T (SCSP-502) were purchased from National Collection of Authenticated Cell Cultures, Chinese Academy of Sciences. All the cells were cultured in Dulbecco’s modified Eagle’s medium (DMEM) supplemented with 10% fetal bovine serum (FBS) and were maintained in a humidified atmosphere of 5% CO_2_ at 37 °C.

### RNA extraction, reverse transcription and quantitative polymerase chain reaction (qPCR)

Total RNA was extracted from clinical samples and cells by TRIzol (Thermo, USA). The first-strand cDNA was generated by PrimeScript™ RT Master Mix (Takara Bio, China). TB Green® Premix Ex Taq™ (Takara Bio, China) was used to perform qPCR reactions according to the manufacturer’s instructions. β-actin was used as endogenous control to normalize the total RNA in each sample. miRNA qPCR was conducted using miRCURY LNA SYBR® Green PCR kit (Qiagen, Germany), and miRNA levels were normalized to U6 levels. The results were calculated using 2^−△△CT^ method. The sequences of all the primers used were listed in Additional file 2: Table S1.

### Lentivirus infection and transient cell transfection

MIR4435-2HG overexpression and control lentiviruses were purchased from GenePharma (Shanghai, China). To obtain MIR4435-2HG overexpression cell lines, cells were cultured in medium added with 5 μg/mL Puromycin after infection. Cells were collected for further experiments at 2 weeks post infection.

All the small interfering RNAs (siRNAs) used in this study were purchased from GenePharma (Shanghai, China). Lipofectamine 3000 (Thermo, USA) was used to transfect siRNA according to the manufacturer’s instruction. The sequences of all the siRNAs used were listed in Additional file 2: Table S1.

### Western blot

Protein samples were extracted by RIPA (Beyotime, China), separated by SDS-PAGE and transferred to NC membranes (Millipore, Germany). After blocked with 5% nonfat milk, the membranes were incubated with primary antibodies against METTL3 (64 kDa, 1:1000, ab195352, Abcam, USA), NOP58 (60 kDa, 1:1000, NBP1-46846, Novus, USA), IGF2BP1 (63 kDa, 1:1000, ab184305, Abcam, USA) or GAPDH (36 kDa, 1:5000, 60004-1-Ig, Proteintech, USA) at 4 °C overnight. After washing for three times, the membranes were incubated with IRdye 700-conjugated goat anti-mouse IgG and IRdye 800-conjugated goat anti-rabbit IgG for 40 min at room temperature. The signals were detected by Odyssey infrared scanner (Li-Cor Biosciences, USA). GAPDH was applied as endogenous control. Raw images from gels or western blots were provided in Additional file 3.

### Immunohistochemistry staining

Immunohistochemistry staining was performed as previously described with a specific anti-NOP58 (1:200, NBP1-46846, Novus) antibody, anti-Ki67 antibody (1:200, 28074-1-AP, Proteintech), or an anti-PCNA (1:200, 10205-2-AP, Proteintech) [14]. Quantification of NOP58 was performed by blindly by pathologist.

### Cell counting kit-8 (CCK-8) assay

3 × 10^3^ cells were seeded into each well of 96-well plates with 100 μL normal culture medium. The experiments were performed using CCK-8 reagent (Dojindo Laboratories, Japan) following the procedures of manufacturer. The value of OD450 was normalized to plot the cell growth curve. All of the experiments were performed in triplicate.

### Colony formation and spheroids formation assay

3 × 10^3^ cells were seeded into each well of 6-well plates with 2 mL normal culture medium and incubated for 10 days. Then fix the cells with 4% polyformaldehyde for 30 min and stain the cells with crystal violet (Beyotime) for 30 min. The colonies in the dishes were photographed and counted.

3 × 10^3^ cells were seeded into each well of 6-well ultra-low attachment culture dishes with 2 mL normal culture medium and incubated for 10 days. Spheroid formation was photographed and assessed by visual inspection under microscope.

### EdU cell proliferation assay

The assay was conducted with EdU kit (RiboBio Co. Ltd, China) according to the instruction. Briefly, incubate the cells with 50 μM EdU A medium at culture environment and fix with 4% polyformaldehyde for 30 min. Then, the cells were stained with 1 × Apollo® for 30 min and the nuclei of cells were stained by DAPI. The total cells (identified by DAPI staining) and the EdU-positive cells (identified by Apollo® fluorescence) were photographed and counted under Olympus BX51 Fluorescence photomicroscope (Olympus, Japan). Each experiment was performed in triplicate.

### Transwell migration and invasion assays

Transwell migration and invasion assays were performed with Millicell hanging Biocoat Matrigel and control chambers from BD Biosciences (24-wellinsert, 8-lm pore size) as previously described [15]. Briefly, 4 × 10^4^ cells in 200 μL serum free medium were loaded into the upper chambers, while lower chambers were filled with 500 μL custom medium. In migration assays, the cells on the underside of the membrane were stained 24 h later. In invasion assays, the cells on the underside of the membrane were stained 48 h later. Then, the cells were counted under a microscope.

### Wound healing assay

Wound healing assays were performed as previously described [16]. Briefly, cells were plated in 6-well plates and incubated at 37 °C. With the cells were complete attached, we scraped the middle of the plate to form a wound and replaced the medium with serum-free medium. After 48 h, the coverage of the line was measured.

### Xenograft tumor model

Five-week-old male BALB/c athymic nude mice were purchased and maintained as previously described [17]. Ethical consent was granted from the Committee on Ethics of Biomedicine, Naval Medical University. Each mouse was injected with 5 × 10^6^ MIR4435-2HG overexpressed Huh7 cells on the left armpit and control on the right armpit (six mice per group). The growth of subcutaneous tumor was recorded each week and the tumor volume was calculated as MIN(a)^2^ × MAX(b) × 0.5. After 6 weeks of observation, the mice were sacrificed and the tumors were obtained, measured, photographed and stored at − 80 °C.

### In vitro limiting dilution assay

The experiments were conducted as previously described with slightly modification [17]. A total of 40, 80, 100, 400, 600, 800 HCC cells with MIR4435-2HG overexpression or control were seeded into 96-well ultra-low attachment culture dishes separately and incubated for 10 days (*n* = 15). Spheroid formation was observed under microscope. The proportion of spheroid-initiation cells was calculated by L-Calc software program (Stem Cell Technologies, Canada) and Poisson’s distribution statistics.

### In vivo limiting dilution assay

A total of 1 × 10^3^, 1 × 10^4^, 1 × 10^5^ and 1 × 10^6^ HCC cells with MIR4435-2HG overexpression or control were injected subcutaneously into male nude mice (*i* = 5). After 8 weeks’ observation, the frequency of tumor-initiating cell was determined by L-Calc software program (Stem Cell Technologies) and Poisson’s distribution statistics.

### RNA pulldown assay

RNA pulldown assay was performed as previously described [13]. Briefly, the biotin labeled MIR4435-2HG, truncated MIR4435-2HG and its antisense RNA were in vitro transcribed from vector pSPT19-MIR4435-2HG by T7 RNA polymerase (Roche, Switzerland) in vitro. Then, incubate the biotin-labeled transcript with Huh7 cell extracts and isolate the RNA–protein complex with Dynabeads Myone Streptavidin T1 beads (Invitrogen, USA). The proteins pulled down were separated by SDS-PAGE, detected by mass spectrum and verified by Western blot.

### Co-Immunoprecipitation (co-IP) assay

RNA pulldown assay was performed as previously described [15]. Briefly, co-IP was performed in Huh7 cells using Pierce™ Co-IP Kit (ThermoFisher Scientific). Immunoprecipitations of NOP58 (NBP1-46846, Novus) or IGF2BP1 (ab184305, Abcam) were performed using an anti-NOP58 or IGF2BP1 antibody overnight at 4 ℃.

### RNA immunoprecipitation (RIP) analysis and methylated RNA immunoprecipitation (MeRIP) analysis

The EZ-Magna RNA-Binding Protein Immunoprecipitation Kit (Millipore) was used to perform RIP and MeRIP assays according to the manufacturer’s instructions. The enriched RNAs were detected by qPCR. Primary antibody against NOP58 (NBP1-46846, Novus), IGF2BP1 (63 ab184305, Abcam) or m6A (A-1801, Epigentek, USA) was used in this study. The sequences of primers were separately listed in Additional file 2: Table S1. These experiments were performed in triplicate.

### Analysis of NOP58 protein stability by CHX and MG132 treatment

Different groups of cells were cultured in 6-well plate and treated with CHX at a concentration of 25 μg/mL. Cell extracts were obtained at specific time points and were further detected by Western blot analysis.

### Reverse transcription at low dNTP concentrations followed by PCR (RTL-P)

The RTL-P approach was conducted as previously described [18]. The key principle is that the 2′-O-methylated nucleotide could impede reverse transcription with low dNTP concentration. The total RNA was extracted and reversely transcribed with specific primers upstream to a methylation site in either a low level (1 μM) or a high level (1 mM) of dNTP (Thermo). The cDNAs were detected by qPCR and the methylation ratios was calculated following the function 2^(CTlow−CThigh)^. The sequences of primers were listed in Additional file 2: Table S1.

### OP-Puro (OPP) incorporation assay

The protein expression rates were detected by Click-iT plus OPP protein synthesis assay kit (Thermo). For initiation, Click-iT OPP was added to culture medium to a final concentration of 20 μM for 30 min. Then, wash the cells with PBS and fix with 4% formaldehyde in PBS for 15 min. After fixation, incubate the cells with 0.5% Triton X-100 in PBS for 15 min. Prepare Click-iT plus OPP reaction cocktail and incubate the cells for 30 min at room temperature. After removing the rinse buffer, stain DNA with DAPI for 30 min. Finally, wash cells twice with PBS, remove the wash solution and proceed to imaging.

### Dual-luciferase assay

The pmirGLO Dual-Luciferase Vector was purchased from GenePharma (Shanghai, China). The wide type 1–1000 bp of MIR4435-2HG and the corresponding mutated were subcloned into the Vector. The pcDNA3-RLuc-PolIRES-FLuc plasmid was kindly provided by Nahum Soneberg [19]. The IRES sequences of insulin like growth factor 1 receptor (IGF1R) and MYC were cloned from Huh7 cells. Dual-luciferase assays were performed following the procedures of manufacturer. The above experiments were conducted in triplicate.

### Statistical analysis

All statistical analyses were performed with SPSS 16.0, Graphpad Prism 8.0 and L-Calc™. Two-tailed Student t test, one-way analysis of variance, and chi-square test were performed for statistical comparison when appropriate. Spearman’s or Pearson’s correlation coefficient was used for statistical correlation. Survival curves were assessed by Kaplan–Meier’s method and log-rank test. *P* values were two-side and a *P* value < 0.05 was considered to be statistically significant. All data were shown as mean ± standard deviation (SD).

## Results

### MIR4435-2HG is a candidate fetal lncRNA in HCC

**Fig. 1:**
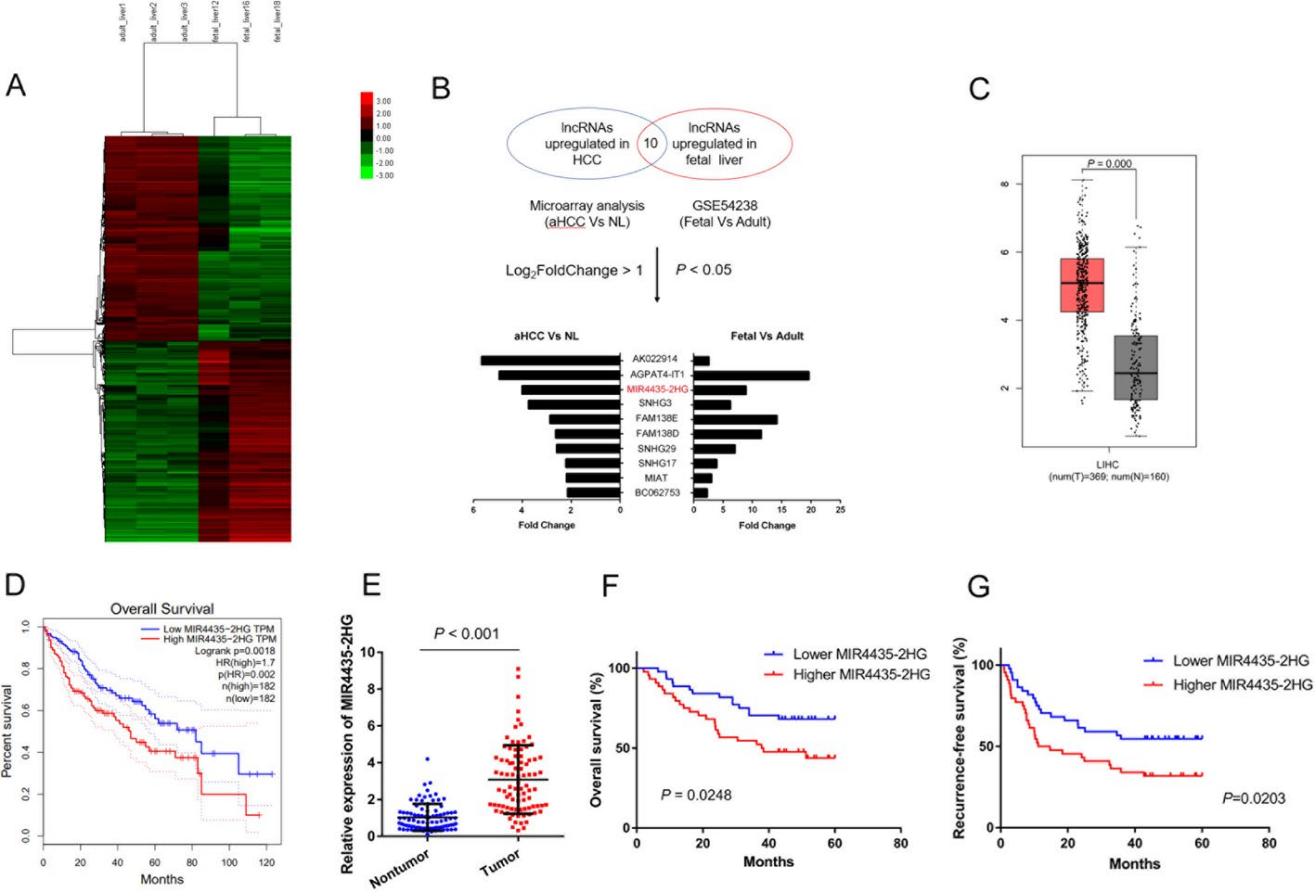
MIR4435-2HG is an oncofetal lncRNA with prognostic value. **A** Heatmaps for lncRNAs that were differently expressed in fetal and adult liver tissues. **B** Schematic representation of screen for oncofetal lncRNAs in HCC and fold changes and *P* values of the overlapped lncRNAs. **C** MIR4435-2HG expression in HCC and nontumor tissues in TCGA-LIHC cohort. **D** Kaplan–Meier’s survival curves of OS in TCGA-LIHC cohort. **E** MIR4435-2HG expression in HCC and paired nontumor tissues in cohort 2 (*n* = 88). **F** and **G** Kaplan–Meier’s survival curves for OS and RFS of HCC patients in cohort 2

To identify oncofetal lncRNAs in HCC, we first analyzed the expression profiles of lncRNAs in 4-months-old human fetal livers (3 samples) and adult livers (3 samples, distal healthy liver tissues extracted from individuals diagnosed with liver hemangioma) using microarray assays. The microarray data are available from Gene Expression Omnibus (GSE225635). The results showed that a total of 4978 lncRNAs were differently expressed (fold-change > 2, *P* value < 0.05), which distinguished fetal liver from adult liver (Fig. 1A). Then, we examined the expression profiles of lncRNAs in HCC and normal liver tissues based on our previous study (GSE54238) [17]. The intersection of the two datasets revealed 10 potential oncofetal lncRNAs that were highly expressed in fetal livers and HCC tissues but turned off in normal adult livers (fold-change > 2, *P* value < 0.05, Fig. 1B).

Among these lncRNAs, MIR4435-2HG, which was in the top three, attracted our attention. With the online bioinformatics tool GEPIA, we found that MIR4435-2HG expression was upregulated in HCC patients from the TCGA-LIHC cohort and elevated MIR4435-2HG expression indicated poor overall survival (Fig. 1C and D; cohort1) [20]. To further clarify the clinical significance of MIR4435-2HG, we detected MIR4435-2HG expression in a cohort containing 88 pairs of HCC and adjacent nontumor tissues (cohort2). The results showed that MIR4435-2HG expression was significantly increased in HCC tissues compared with nontumor tissues (Fig. 1E). Kaplan–Meier analysis revealed that high MIR4435-2HG expression in HCC tissues was correlated with increased recurrence and poor overall survival (Fig. 1F and G). Moreover, correlation regression analysis showed that high MIR4435-2HG expression was correlated with large tumor size, aggressive TNM stages, and microvascular invasion (Table 1). Thus, we focused on MIR4435-2HG to elucidate its role in HCC.

**Table 1:** Clinical characteristic of HCC patients

Feature	MIR4435-2HG		Chi-square	*p*-value
	Low	High		
Gender			0.34	0.772
Male	36	38		
Female	8	6		
Age			0.414	0.668
< 50	26	23		
≥ 50	18	21		
AFP			0.196	0.825
≤ 20 µg/L	15	17		
> 20 µg/L	29	27		
Differentiation			0.279	0.792
Well	10	8		
Poorly	34	36		
TNM			5.057	0.042^*^
I	20	10		
II/III	24	34		
BCLC			5.437	0.036^*^
A	14	5		
B or C	30	39		
Liver cirrhosis			0.218	0.816
No	12	14		
Yes	32	30		
Tumor diameter			5.503	0.032^*^
< 5 cm	27	16		
≥ 5 cm	17	28		
Tumor encapsulation			0.182	0.813
None or incomplete	22	20		
Complete	22	24		
MIT			5.526	0.032^*^
None	29	18		
Yes	15	26		

### MIR4435-2HG promotes the progression of HCC

**Fig. 2:**
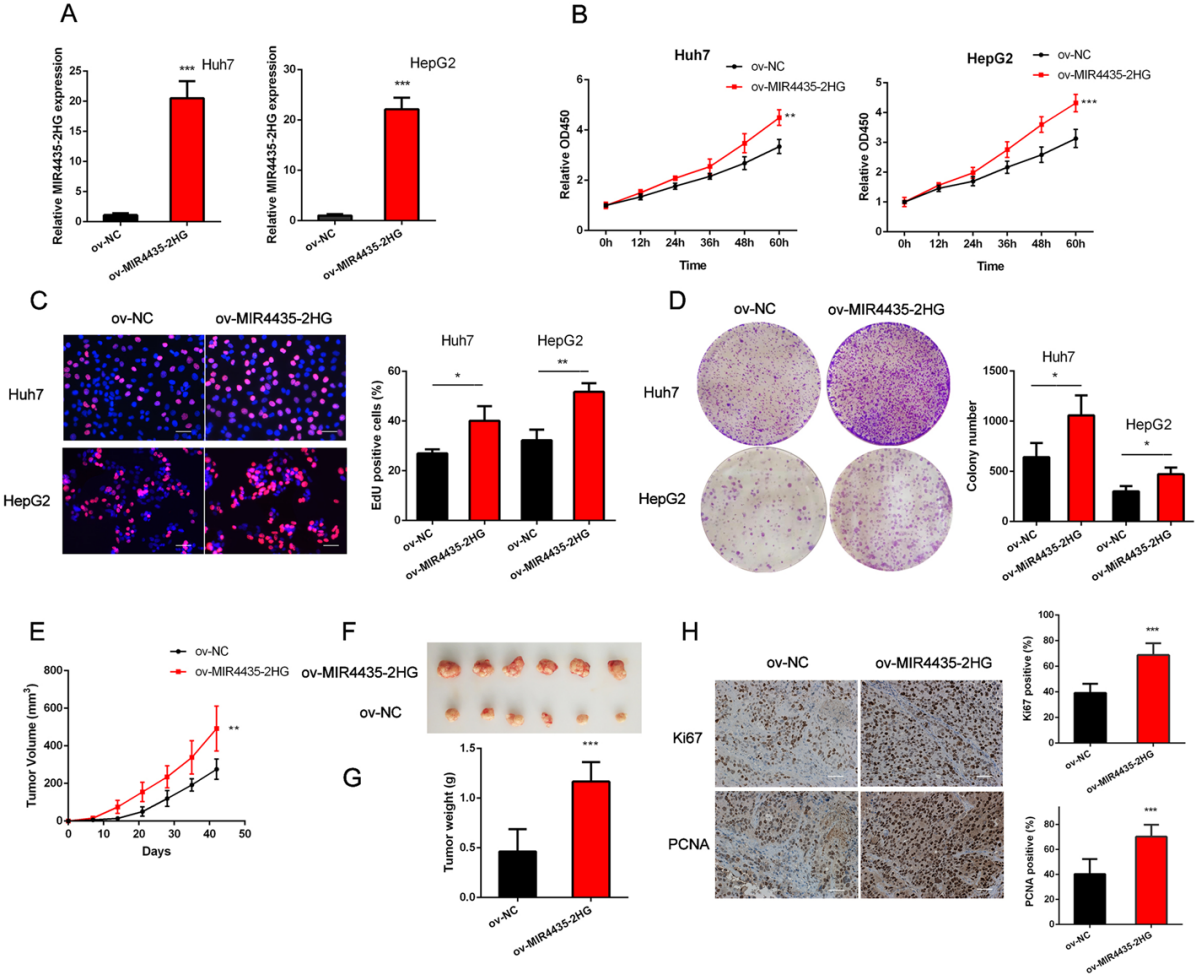
MIR4435-2HG promotes the progression of HCC cells in vitro and in vivo. **A** MIR4435-2HG expression in HCC cells with lentivirus infection. **B** Cellular proliferation of HCC cells with MIR4435-2HG overexpression or control was detected by CCK8 assay. **C** Cellular proliferation of HCC cells with MIR4435-2HG overexpression or control was detected by EdU assay. **D** Colony formation ability of HCC cells with MIR4435-2HG overexpressed or control was detected. **E** Tumor volume grow curves of subcutaneous tumor model. **F** Weight of subcutaneous tumors formed by Huh7 cell with MIR4435-2HG overexpression or control. **G** Photograph of subcutaneous tumors formed by Huh7 cell with MIR4435-2HG overexpression or control. **H** Representative images of Ki-67 and PCNA immunohistochemical staining in tumor tissues. The data are shown as mean ± SD. ^*^P < 0.05, ^**^P < 0.01, ^***^P < 0.001. Scale bar, 50 μm

To explore the biological function of MIR4435-2HG in hepatocarcinogenesis, we stably overexpressed MIR4435-2HG in HepG2 and Huh7 cells with lentivirus infection, and knocked down MIR4435-2HG expression with siRNA transfection (Fig. 2A and Additional file 1: Fig. S1A). As indicated by CCK8 assays and EdU staining, overexpression of MIR4435-2HG strikingly increased HCC cell proliferation (Fig. 2B and C), while silencing MIR4435-2HG inhibited growth of HCC cells (Additional file 1: Fig. S1B and C). Meanwhile, MIR4435-2HG overexpression accelerated colony formation of HCC cells (Fig. 2D); however, knockdown of MIR4435-2HG led to decreased colony formation (Additional file 1: Fig. S1D). Although we conducted transwell migration and invasion assays as well as wound healing assays, the findings indicated that depletion of MIR4435-2HG did not exert an impact on the migratory and invasive capacities of HCC cells (Additional file 1: Fig. S1E-G). Collectively, the above results showed that MIR4435-2HG could promote the proliferation of HCC cells in vitro.

To verify the role of MIR4435-2HG in hepatocarcinogenesis in vivo, Huh7 cells with MIR4435-2HG overexpression and control were subcutaneously injected in the left and right flank of athymic nude mice separately. Consistent with the in vitro results, 6 weeks of observation showed that overexpression of MIR4435-2HG could accelerate tumor growth (Fig. 2E). Further measurements revealed that xenografts derived from Huh7 cells with MIR4435-2HG overexpression developed larger volumes and higher weights than that from control (Fig. 2F and G). In addition, higher Ki67 and PCNA rate was detected in MIR4435-2HG overexpression group (Fig. 2H). Altogether, these results showed that MIR4435-2HG could enhance HCC proliferation in vivo.

### MIR4435-2HG enhances the stemness features of HCC

**Fig. 3:**
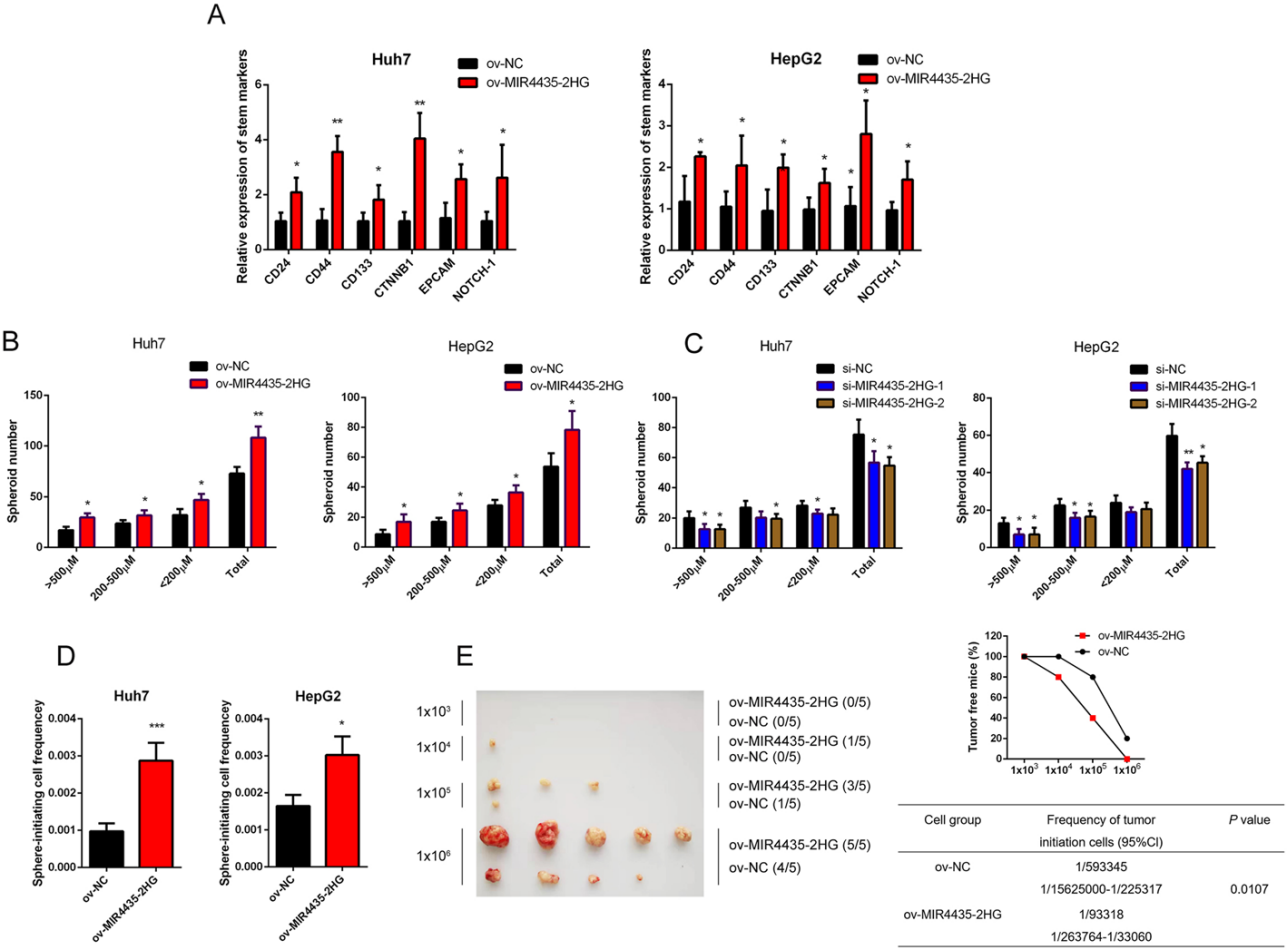
MIR4435-2HG enhances the stemness properties of HCC cells in vitro and in vivo. **A** Expression of stemness markers in HCC cells with MIR4435-2HG overexpression or control was detected by qPCR. **B** Expression of stemness markers in HCC cells with MIR4435-2HG overexpression or control was detected by western blot. **C** The spheroids form ability of HCC cells with MIR4435-2HG overexpression. **D** The spheroids form ability of HCC cells with MIR4435-2HG interference. **E** Overexpression of MIR4435-2HG could significantly enhance the sphere-initiating cell frequency in HCC cell in vitro. **F** Overexpression of MIR4435-2HG could significantly enhance the sphere-initiating cell frequency in HCC cell in vivo. The data are shown as mean ± SD. ^*^P < 0.05, ^**^P < 0.01, ^***^P < 0.001

Previous studies have indicated that oncofetal genes might serve as a link between stem cell maintenance in cancer progression and embryonic development [21]. Thus, we wondered that whether MIR4435-2HG was required in stemness acquisition in HCC. In a panel of 20 HCC samples from cohort2, we found that there was a positive correlation between the expression of MIR4435-2HG and the well-known liver stem cell markers, including EpCAM, CD24 and CD44 (Additional file 1: Fig. S2A–C). This observation was further confirmed in HCC samples from the TCGA-LIHC cohort (Additional file 1: Fig. S2D and E). Furthermore, qPCR showed that overexpression of MIR4435-2HG increased the stem cell markers expression in HCC cells (Fig. 3A).

It has been proposed that tumor cells with stemness properties play crucial roles in tumor initiation and growth [22]. In light of this, we detected the sphere formation ability of HCC cells in vitro. The results showed that HCC cells with MIR4435-2HG overexpression formed more and larger tumor spheroids compared with controls (Fig. 3B, Additional file 1: Fig. S3A). Conversely, the sphere formation ability of HCC cells decreased with MIR4435-2HG silencing (Fig. 3C, Additional file 1: Fig. S3B). Furthermore, in vitro limiting dilution assays showed that overexpression of MIR4435-2HG led to an increase in sphere-initiating cells (Fig. 3D). Consistently, in vivo limiting dilution assays confirmed that the volume and growth of HCC cells was significantly increased by MIR4435-2HG overexpression (Fig. 3E). Above all, upregulation of MIR4435-2HG might strengthen the stemness features and drive the oncofetal reprogramming of HCC cells.

### MIR4435-2HG directly binds to NOP58 and IGF2BP3

**Fig. 4:**
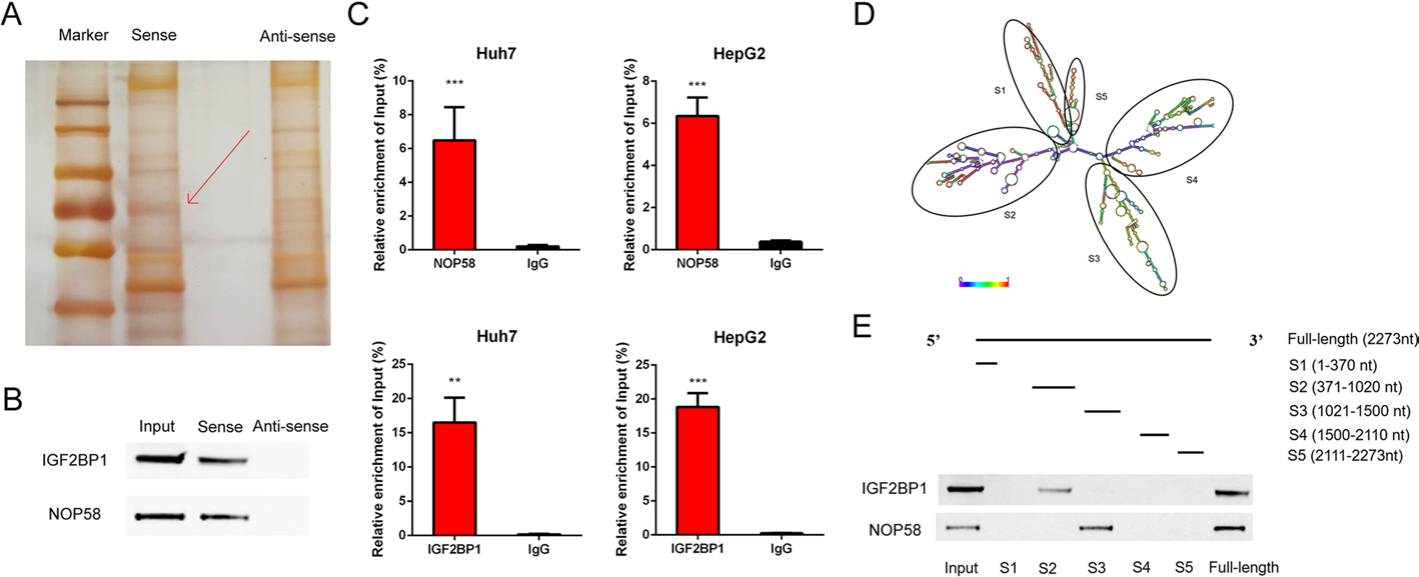
MIR4435-2HG directly binds to NOP58 and IGF2BP1. **A** The silver-stained SDS-PAGE gel of proteins pulled down by MIR4435-2HG sense and antisense RNAs. **B** Western blot analysis showed that NOP58 and IGF2BP1 were in the proteins pulled down by MIR4435-2HG. **C** RIP-PCR assay was performed to enrich MIR4435-2HC by antibody against NOP58 or IGF2BP1 in extracts from HCC cells. **D** The predicted secondary structure of MIR4435-2HG. **E** Serial deletions of MIR4435-2HG were constructed to identify the specific association of MIR4435-2HG and NOP58 or IGF2BP1

To elucidate the mechanisms underlying the oncogenic role of MIR4435-2HG in HCC, we performed RNA Pulldown assay and identified that the molecular weight of MIR4435-2HG-associated protein should be around 60 kDa (Fig. 4A). Subsequent mass spectrometric analysis and western blot revealed that IGF2BP1 and NOP58 specifically bound to MIR4435-2HG (Fig. 4B, Additional file 2: Table S2). Meanwhile, RIP assays showed that MIR4435-2HG was enriched to the antibodies against IGF2BP1 and NOP58 (Fig. 4C).

To characterize the interaction between MIR4435-2HG and NOP58, as well as between MIR4435-2HG and IGF2BP1, we constructed a series deletion of MIR4435-2HG base on the secondary structure prediction (http://rna.tbi.univie.ac.at/cgi-bin/RNAWebSuite/RNAfold.cgi, Fig. 4D). Subsequently, western blot analysis showed that 1021–1500-nt fragments of MIR4435-2HG were necessary for the interaction with NOP58 (Fig. 4E), while 371–1020-nt were necessary for the interaction between with IGF2BP1 (Fig. 4E). Further co-IP assays unveiled the absence of a direct interaction between IGF2BP1 and NOP58 (Additional file 1: Fig. S4).

### M6A mediated the upregulation of MIR4435-2HG in HCC

**Fig. 5:**
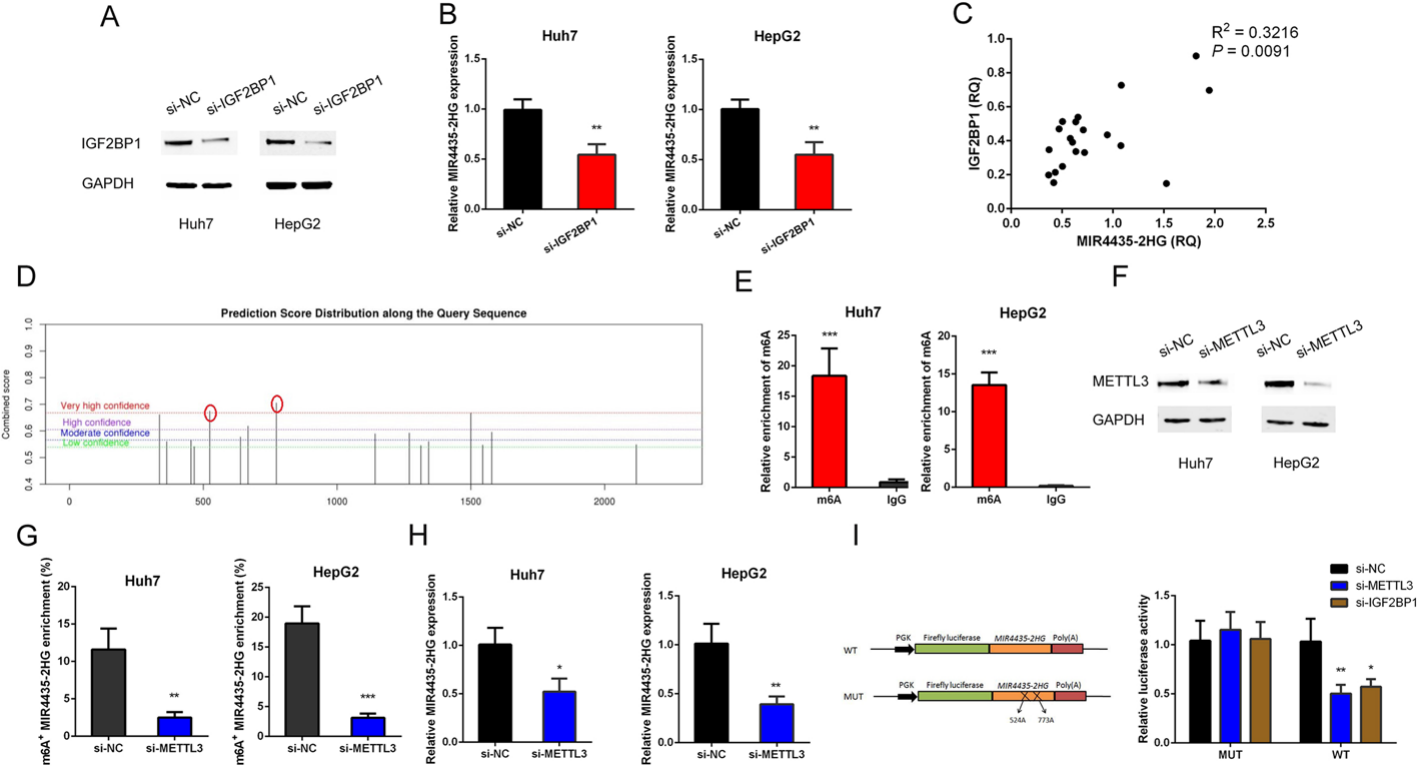
IGF2BP1 regulates MIR4435-2HG expression in a m6A dependent way. **A** Knockdown of IGF2BP1 by siRNA in HCC cells. **B** The expression of MIR4435-2HG in HCC cells with IGF2BP1 knockdown. **C** The relationship between MIR4435-2HG expression and IGF2BP1 expression in 20 HCC samples. **D** The predicted conserved m6A modification sites in MIR4435-2HG. **E** MeRIP-PCR analysis was performed to confirm the enrichment of m6A modification in MIR4435-2HG in HCC cells. **F** Knockdown of METTL3 by siRNA in HCC cells. **G** The enrichment of m6A modification in MIR4435-2HG in HCC cells with METTL3 knockdown. **H** The expression of MIR4435-2HG in HCC cells with METTL3 knockdown. **I** The dual-luciferase reporter assay showed that METTL3 and IGF2BP1 regulated MIR4435-2HG expression in a m6A dependent. The data are shown as mean ± SD. ^*^P < 0.05, ^**^P < 0.01, ^***^P < 0.001

To further explore the biological function of IGF2BP1-MIR4435-2HG interaction, we detected the expression of IGF2BP1 with MIR4435-2HG overexpressed. The result showed that MIR4435-2HG did not affect the expression of IGF2BP1 in both mRNA and protein level (Additional file 1: Fig. S5A and B). However, knockdown of IGF2BP1 decreased the MIR4435-2HG expression in HCC cells (Fig. 5A and B). Meanwhile, a correlation between the expression of IGF2BP1 and MIR4435-2HG was observed in 20 HCC tissues from cohort2 (Fig. 5C). It has been widely acknowledged that IGF2BP1 is a oncofetal gene, as well as an important m6A “reader”. According to an online m6A site predictor (SRAMP, http://www.cuilab.cn/sramp), there were two highly conserved m6A sites in the IGF2BP1 binding fragment of MIR4435-2HG (Fig. 5D). Subsequent methylated RNA immunoprecipitation (MeRIP) assay verified that MIR4435-2HG bore m6A modification in HCC cells (Fig. 5E). Considering the important role of m6A modification in embryonic development and tumorigenesis, we proposed that IGF2BP1 might regulate MIR4435-2HG expression in a m6A dependent way.

The dynamic m6A modifications are primarily regulated by m6A methyltransferase and demethylase. Thus, we decreased the total m6A level in HCC cells with knockdown of METTL3, which serves as a m6A methyltransferase and an oncogene in HCC (Fig. 5F). With immunoprecipitated m6A from RNAs of METTL3 knockdown and control cells, we found that the amount of MIR4435-2HG modified by m6a was substantially decreased by METTL3 interference (Fig. 5G). Further qPCR analysis showed that METTL3 knockdown led to decreased expression of MIR4435-2HG in HCC cells (Fig. 5H). To verify the predicted m6A modification sites, we performed the dual-luciferase reporter assays. The results showed that knockdown of IGF2BP1 or METTL3 decreased the luciferase activity, while mutation of m6A modification sites within MIR4435-2HG abrogated the suppression of luciferase activity induced by IGF2BP1or METTL3 interference (Fig. 5I). These data demonstrated that IGF2BP1 enhanced expression of MIR4435-2HG in HCC through m6A modification.

### MIR4435-2HG contributes to progression and stemness of HCC via stabilizing NOP58

**Fig. 6:**
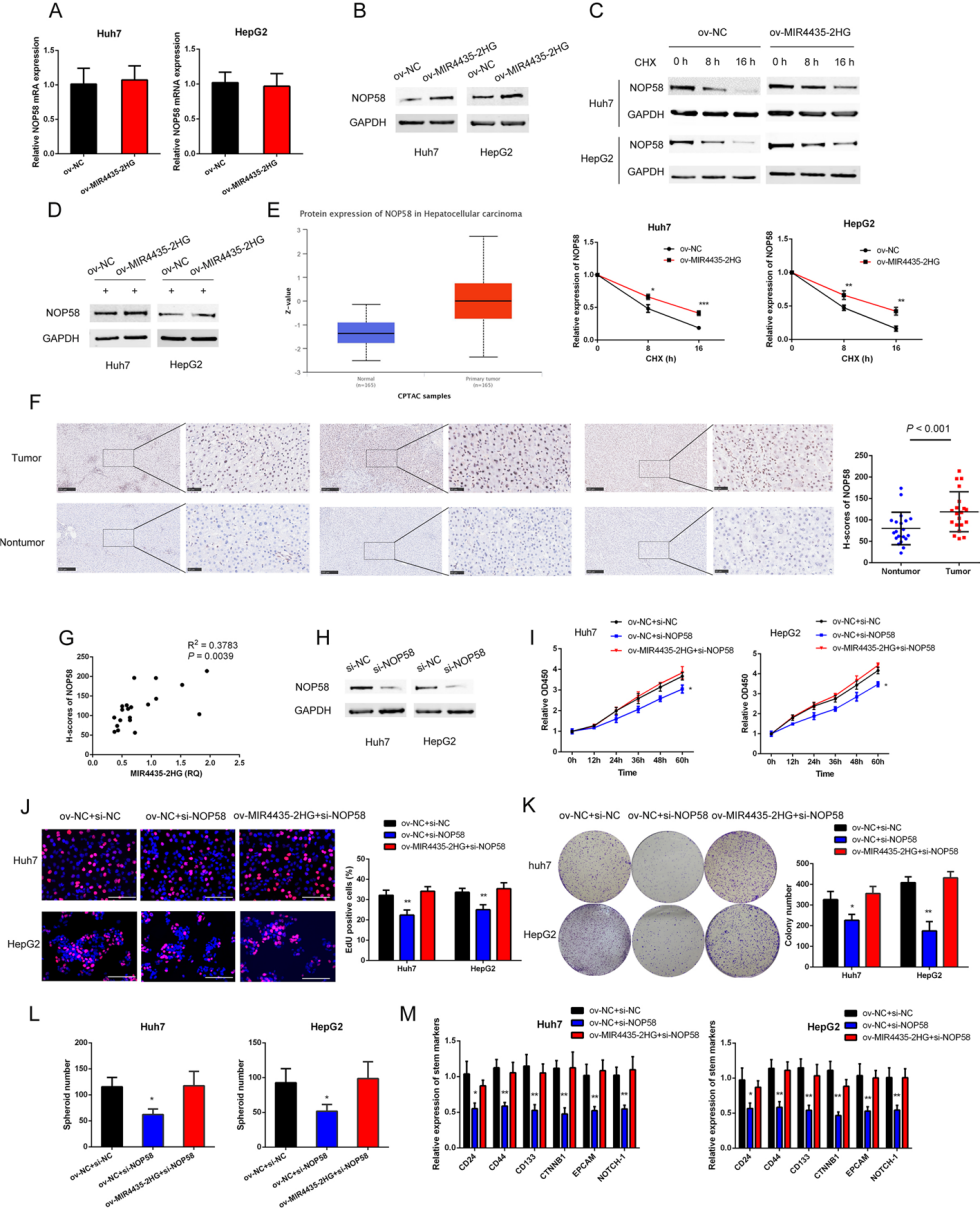
NOP58 was stabilized by MIR4435-2HG and mediated the pro-tumorigenetic role of MIR4435-2HG in HCC. **A** The expression of NOP58 mRNA in HCC cells with MIR4435-2HG interference in HCC cells. **B** The expression of NOP58 protein in HCC cells with MIR4435-2HG overexpressed in HCC cells. **C** Western blot analysis detected the expression of NOP58 protein in HCC cells with treatment of CHX. **D** Western blot analysis detected the expression of NOP58 protein in HCC cells with treatment of MG132. **E** Expression analysis of NOP58 protein in HCC based on CPTAC database. **F** Representative images of the expression of NOP58 protein in paired tumor and nontumor tissues by IHC. **G** The relationship between MIR4435-2HG expression and NOP58 expression in 20 HCC samples. H Knockdown of NOP58 by siRNA in HCC cells. **I** Cellular proliferation of HCC cells was detected by CCK8 assay. **J** Cellular proliferation of HCC cells was detected by EdU assay. **K** Colony formation ability of HCC cells was detected. **L** The spheroids form ability of HCC cells. **M** Expression of stem cell markers were detected in the indicated groups by qPCR. The data are shown as mean ± SD. ^*^P < 0.05, ^**^P < 0.01, ^***^P < 0.001. White scale bar, 100 μm

Then, we sought to detect the molecular consequence of NOP58-MIR4435-2HG interaction. The results showed that MIR4435-2HG overexpression significantly increased the total amount of NOP58 protein (Fig. 6B), but not NOP58 mRNA level (Fig. 6A). We further demonstrated that overexpression of MIR4435-2HG extended the half-life of NOP58 protein with CHX treatment, suggesting MIR4435-2HG did not affect protein synthesis of NOP58 (Fig. 6C). However, with the treatment of the proteasome inhibitor MG132, the increasement of NOP58 accumulation following MIR4435-2HG overexpression was abrogated (Fig. 6D). Taken together, these data indicated that MIR4435-2HG directly binds to NOP58 and stabilizes NOP58 through protecting it from degradation.

With the online bioinformatics tool UALCAN, we analyzed NOP58 expression based on data from Clinical Proteomic Tumor Analysis Consortium (CPTAC) datasets and found that the expression of NOP58 protein was significantly upregulated in HCC tissues compared with normal liver tissues (Fig. 6E). Meanwhile, we also confirmed upregulation of NOP58 in HCC tissues with immunohistochemical (IHC) assays in 20 paired tumor and nontumor tissues from cohort2 (Fig. 6F). Moreover, NOP58 and MIR4435-2HG levels were positively corelated within HCC tissues (Fig. 6G). To gain insights into the role of NOP58 in tumorigenesis, as well as pro-tumorigenic effects of MIR4435-2HG, we inhibited NOP58 expression in HCC cells by siRNA (Fig. 6H). The results showed that NOP58 knockdown reduced proliferation, DNA replication, and survival of HCC cells. Additionally, NOP58 knockdown partially reserved the effects of MIR4435-2HG overexpression on cell proliferation, DNA replication, and survival (Fig. 6I–K). We further detected the role of NOP58 in MIR4435-2HG mediated stem cell maintenance of HCC cells. Our results showed that NOP58 interference abrogated the increased spheroid formation ability of HCC cells with MIR4435-2HG overexpressed (Fig. 6L and Additional file 1: Fig. S6). Consistently, decreased expression of stem cell markers induced by NOP58 interference could be rescued by MIR4435-2HG overexpression (Fig. 6M). Collectively, these data demonstrated that NOP58 mediated the oncogenic roles of MIR4435-2HG in HCC.

### MIR4435-2HG/NOP58 regulates rRNA 2′-O-Me and IRES-dependent translation of oncogene in HCC

**Fig. 7:**
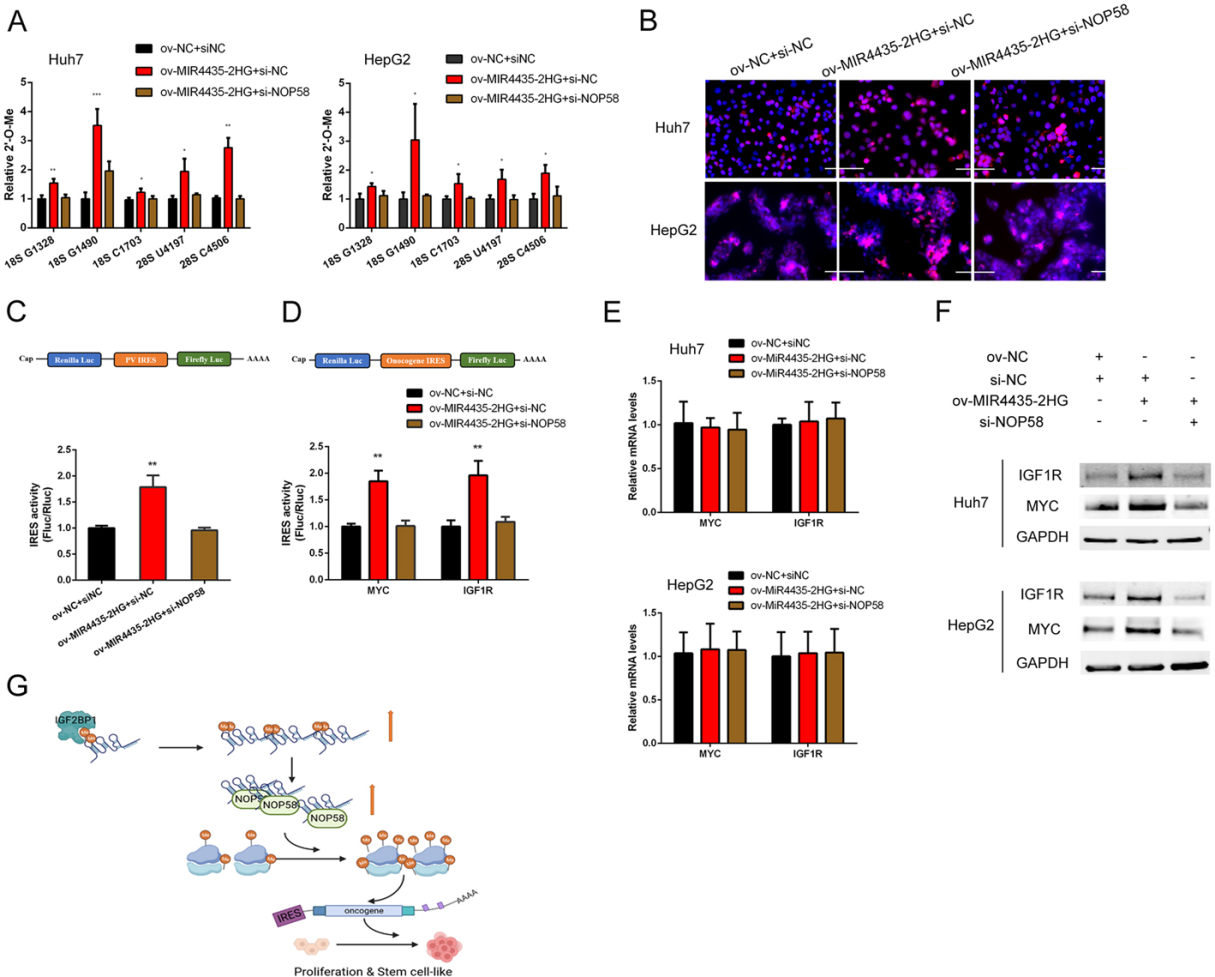
Overexpression of MIR4435-2HG increases rRNA 2′f-O methylation and IRES-dependent translation of oncogene in HCC. **A** RTL-P assay was conducted to detect the 2’-O methylation level of rRNA in HCC cells with MIR4435-2HG overexpression or NOP58 knockdown. **B** OP-Puro incorporation assay was conducted to detect the protein synthesis rate. **C** The bi-cistronic luciferase reporter was constructed as above and the Poliovirus (PV) IRES activity was calculated as the ratio of firefly luciferase activity over renilla luciferase activity. **D** The bi-cistronic luciferase reporter was constructed as above and the IRES-dependent translation (Fluc/Rluc) from IRES elements of MYC and IGF1R was measured. **E** qPCR analysis detected the expression of MYC and IGF1R mRNA in the indicated groups. **F** The protein expression of MYC and IGF1R in the indicated groups was detected by western blot analysis. **G** Model of m6A modified MIR4435-2HG regulating stemness maintenance of HCC by stabilizing NOP58. The data are shown as mean ± SD. ^*^P < 0.05, ^**^P < 0.01, ^***^P < 0.001. Scale bar, 100 μm

Given that NOP58 serves as a key component of box C/D small nucleolar ribonucleoproteins (snoRNPs), we postulated whether NOP58-MIR4435-2HG interaction could affect rRNA 2’-O-Me and protein synthesis. As indicated by RTL-P assay, overexpression of MIR4435-2HG significantly increased the 2′-O-Me levels in key sites of rRNA, while NOP58 knockdown partially reserved the effects of MIR4435-2HG overexpression (Fig. 7A). With OP-Puro incorporation assay, we found that overexpression of MIR4435-2HG accelerated protein synthesis rate and NOP58 interference had opposite effects (Fig. 7B). Previous study has indicated that 2′-O-Me is responsible for IRES-dependent mode of translation initiation [23]. Thus, dual-luciferase reporter with Poliovirus (PV) IRES-driven firefly luciferase (Fluc) was constructed. Upon overexpression of MIR4435-2HG, the Fluc/Rluc ratio, which indicates IRES-dependent translation, was significantly increased. In contrast, knockdown of NOP58 repressed translation efficiency (Fig. 7C). Among genes with IRESs identified in their 5’UTR, we took an interest in MYC and IGF1R, which are closely associated with cancer stem cell properties. IRES sequences from MYC and IGF1R mRNA in Huch7 cells were cloned and inserted into bi-cistronic luciferase vector separately. As expected, IRESs of MYC and IGF1R showed significantly increased activities with MIR4435-2HG overexpression, while NOP58 interference reduced the activities (Fig. 7D). Western blot and qPCR analysis further confirmed that MIR4435-2HG/NOP58 regulated the translation of MYC and IGF1R in HCC cells (Fig. 7E and F). Thus, NOP58-MIR4435-2HG contributed to the proliferation and stem cell like property of HCC cells by regulating rRNA 2′-O-Me and IRES-dependent translation (Fig. 7G).

## Discussion

Oncofetal genes have shown unique expression patterns in aggressive tumor cells and embryonic progenitors, thereby indicting their potential value in cancer diagnosis and treatment [24]. In this study, we identified MIR4435-2HG as an oncofetal lncRNA. Clinical studies revealed that MIR4435-2HG levels were associated with tumor recurrence and cancer-related death of HCC patients. With in vitro and in vivo methods, we also confirmed that overexpression of MIR4435-2HG promotes the progression and stem properties of HCC cells.

Numerous studies suggest that the complicated crosstalk between miRNAs and lncRNAs plays important roles in cancer progression. Besides function as miRNA sponges, lncRNAs may also serve as host genes for miRNAs [25]. The relationship between miRNAs and their hosting lncRNAs can be either antagonistic or synergistic [26, 27]. In this study, MIR4435-2HG serves as a miRNA host lncRNA with miR-4435-2 located on its sense strand. To explore the relationship between MIR4435-2HG and miR-4435-2 in HCC, we detected miR-4435-2 expression in 45 pairs of HCC tissues and paired nontumor tissues from cohort 2. The results showed that there was no significant difference in miR-4435-2 expression between HCC tissues and paired nontumor tissues, and the expression of MIR4435-2HG and miR-4435-2 did not correlate in HCC tissues (Additional file 1: Fig. S7A and B). Thus, miR-4435-2 and its hosting lncRNA MIR4435-2HG may exert independent function in HCC.

In addition to governing initiation and propagation, the stemness features confer cancer cells with functional heterogeneity and therapy resistance. Given the important role of MIR4435-2HG in stemness maintenance of HCC cells, we further explored whether MIR4435-2HG is involved in inherent and acquired resistance of Lenvatinib, the first-line chemotherapeutic therapy for advanced HCC. We found that overexpression of MIR4435-2HG increased the half maximal inhibitory concentration (IC50) values for Lenvatinib (Additional file 1: Fig. S8A). Meanwhile, colony formation assays also revealed that MIR4435-2HG overexpression significantly reduced the cell susceptibility to Lenvatinib (Additional file 1: Fig. S8B). However, further in vivo* experiments* should be conducted to characterize the role of MIR4435-2HG in acquisition of Lenvatinib resistance.

Though the value of oncofetal genes as biomarkers has been well appreciated, the precise mechanism that drives re-expression of oncofetal gene in cancer remains unclear. Aberrant methylation in the promoter region of oncofetal genes has been observed in various types of cancer. In the case of HCC, DNA demethylation has been reported to reactive the expression of SALL4 [28]. However, the promoter region of MIR4435-2HG bared no DNA methylation (Additional file 1: Fig. S9). Chemical modifications in RNA, similar to those in DNA and core histone, have been found to play important roles in regulating gene expression [29]. Among these modifications, m6A, the most abundant internal mRNA and lncRNA modification in higher eukaryotes, has received great attention recently. Accumulating evidence suggests that m6A is involve in multiple cellular processes by regulating gene expression [30]. Here, we revealed that upregulation of MIR4435-2HG was mediated by IGF2BP1 in a m6A-dependent way. Meanwhile, knockdown of IGF2BP1 decreased the expression of NOP58 in HCC (Additional file 1: Fig. S10). IGF2BP1 is a well-known m6A reader, as well as an oncofetal gene [31, 32]. In HCC, IGF2BP1 directly bound to MIR4435-2HG and regulated the expression of MIR4435-2HG/NOP58. However, whether m6A is involved in the upregulation of other oncofetal genes are largely unknown. Moreover, changes in the distribution of m6A in RNA during embryonic development and tumorigenesis remain elusive. Further transcriptome-wide m6A-seq analysis may provide more insights into the role of m6A modification in oncofetal gene reactivation and tumorigenesis.

With RNA pulldown assay, we found that MIR44435-2HG also bound to NOP58 and stabilized it. NOP58 is a core factor in the assembly box C/D snoRNA/RNP that mediates 2′-O-Me modification network [33]. In this study, we found that the upregulation of NOP58 increased 2’-O-Me levels of rRNA in HCC cells. As the most abundant rRNA modification, Ribose 2′-O-Me shows a complex pattern in eukaryotic cells. Recent advances in 2′-O-Me quantification and ribosome profiling revealed that 2′-O-Me is required in rRNA processing and secondary structure formation, and plays an important role in ribosome biogenesis, proper gene expression and protein synthesis [34]. Changes in 2′-O-Me modification may alter cellular behavior and increase cancer susceptibilities. Indeed, overexpression of FBL, another component of box C/D snoRNA/RNP, was observed in multiple cancers and contributed to tumorigenesis through altering translation regulation [35]. Box snoRNA/RNP formation was reported to be essential in leukaemic stem cell self-renewal and leukaemogenesis [36, 37]. Our study showed that MIR4435-2HG mediated upregulation of NOP58 contributed to stem cell-like properties and progression of HCC cells by accelerating IRES-dependent translation of oncogenes, including IGF1R and MYC. Based on these observations, we offered a better understanding of the role of lncRNA in 2′-O-Me-dependent translation regulation and the involvement of post-transcriptional RNA in HCC (Additional file 3).

## Conclusions

Taken together, we identified an oncofetal lncRNA MIR4435-2HG, which was upregulated by IGF2BP1 in a m6A-dependent way, and elucidated the function of MIR4435-2HG/NOP58 axis in stemness maintenance and progression of HCC. We characterized the specific role of lncRNA-dependent 2′-O-Me regulation in fetal-like reprograming of HCC cells, provided new insights into oncofetal gene reactivation and revealed the prognostic and therapeutic value of MIR4435-2HG/NOP58 axis for HCC.

### Supplementary Information

**Additional file 1: Fig. S1.** Knockdown of MIR4435-2HG decreases the progression of HCC cells. **A** MIR4435-2HG expression in HCC cells with siRNA transfection. **B** Cellular proliferation of HCC cells with MIR4435-2HG interference or control was detected by CCK8 assay. **C** Cellular proliferation of HCC cells with MIR4435-2HG interference or control was detected by EdU assay. **D** Colony formation ability of HCC cells with MIR4435-2HG interference or control was detected. The data are shown as mean ± SD. **E** Migration abilities of HCC cells with MIR4435-2HG interference or control was detected by transwell migration assay. **F** Invasion abilities of HCC cells with MIR4435-2HG interference or control was detected by transwell invasion assay. **G** Migration abilities of HCC cells with MIR4435-2HG interference or control was detected by wound healing assay. ^*^P < 0.05, ^**^P < 0.01. Scale bar, 50 μm. **Fig. S2.** The relationship between the expression of MIR4435-2HG and stem cell markers. **A** Correlation analysis between expression of MIR4435-2HG and EPCAM in cohort2. **B** Correlation analysis between expression of MIR4435-2HG and CD44 in cohort2. **C** Correlation analysis between expression of MIR4435-2HG and CD133 in cohort2. **D** Correlation analysis between expression of MIR4435-2HG and CD24 in TCGA-LIHC cohort. **E** Correlation analysis between expression of MIR4435-2HG and CD44 in TCGA-LIHC cohort. **Fig. S3.** MIR4435-2HG contributes to the sphere formation ability of HCC cells. **A** Representative pictures of tumor spheroids formed with MIR4435-2HG overexpression or control. **B** Representative pictures of tumor spheroids formed with MIR4435-2HG interference or control. Scale bar, 50 μm. **Fig. S4.** Co-IP was conducted to detect the interaction between NOP58 and IGF2BP1 in Huh7. **Fig. S5.** MIR4435-2HG dose not affect the expression of IGF2BP1 in HCC cells. **A** Overexpression of MIR4435-2HG did not affect mRNA levels of IGF2BP1 in HCC cells. **B** Overexpression of MIR4435-2HG did not affect protein levels of IGF2BP1 in HCC cells. **Fig. S6.** Representative pictures of tumor spheroids formed with different treatment. Scale bar, 50 μm. **Fig. S7.** The role of mir4435-2 in HCC. A Relative expression of mir4435-2 in HCC and pair nontumor tissues. B The relationship between expression of mir4435-2 and MIR4435-2HG in HCC. **Fig. S8.** The role of MIR4435-2HG in lenvatinib resistance. A The IC50 value of Lenvatinib was measured in MIR4435-2HG overexpression and control HCC cells. B Colony formation assays were used to detect the sensitivity of HCC cells to Lenvatinib. ^***^P < 0.001. **Fig. S9.** The predicted DNA methylation modification in the promoter region of MIR4435-2HG by Methprimer. **Fig. S10.** Knockdown of IGF2BP1 decreased the expression of NOP58 in HCC cells. **Fig. S11.** Expression of AGPAT4-IT1 in HCC patients from the TCGA-LIHC cohort. A Expression of AGPAT4-IT1 in HCC and nontumor tissues in TCGA-LIHC cohort. B Kaplan–Meier’s survival curves of OS in TCGA-LIHC cohort.

**Additional file 2: Table S1.** Sequences of primers for qPCR and RTL-P and siRNA used in this study. **Table S2.** Mass spectrometry analysis of the gel.

**Additional file 3: **Raw images from gels or western blots.

## Data Availability

All data generated in this investigation are available from the corresponding author on reasonable request. The datasets generated during lncRNA microarray analysis are available in GEO with accession number GSE225635.

## Abbreviations

lncRNA: Long non-coding RNA
HCC: Hepatocellular carcinoma
NOP58: NOP58 ribonucleoprotein
IGF2BP1: Insulin like growth factor 2 mRNA binding protein 1
IGF1R: Insulin like growth factor 1 receptor
2′-O-Me: 2′-O-methylation
RTL-P: Low dNTP concentrations followed by PCR
RIP: RNA immunoprecipitation
MeRIP: Methylated RNA immunoprecipitation
m6A: N6-methyladenosine
IRES: Internal ribosome entry site
snoRNPs: Small nucleolar ribonucleoproteins
PV: Poliovirus
Fluc: Firefly luciferase
DMEM: Dulbecco’s modified Eagle’s medium
FBS: Fetal bovine serum
qPCR: Quantitative polymerase chain reaction
siRNA: Small interfering RNA
IHC: Immunohistochemistry
CCK-8: Cell counting kit-8
OPP: O-Propargyl-Puromycin
GEO: Gene expression omnibus
TCGA: The Cancer Genome Atlas
CPTAC: Clinical Proteomic Tumor Analysis Consortium
RNP: Small nucleolar ribonucleoproteins
