# Supplementary material for: N6-methyladenosine-modified oncofetal lncRNA MIR4435-2HG contributed to stemness features of hepatocellular carcinoma cells by regulating rRNA 2′-O methylation
Source: Cell Mol Biol Lett. 2023 Oct 27;28:89. doi: 10.1186/s11658-023-00493-2 (PMC10612268; doi:10.1186/s11658-023-00493-2)
Supplement: Supplementary file 1 — Additional file 1: Fig. S1. Knockdown of MIR4435-2HG decreases the progression of HCC cells. A MIR4435-2HG expression in HCC cells with siRNA transfection. B Cellular proliferation of HCC cells with MIR4435-2HG interference or control was detected by CCK8 assay. C Cellular proliferation of HCC cells with MIR4435-2HG interference or control was detected by EdU assay. D Colony formation ability of HCC cells with MIR4435-2HG interference or control was detected. The data are shown as mean ± SD. E Migration abilities of HCC cells with MIR4435-2HG interference or control was detected by transwell migration assay. F Invasion abilities of HCC cells with MIR4435-2HG interference or control was detected by transwell invasion assay. G Migration abilities of HCC cells with MIR4435-2HG interference or control was detected by wound healing assay. *P < 0.05, **P < 0.01. Scale bar, 50 μm. Fig. S2. The relationship between the expression of MIR4435-2HG and stem cell markers. A Correlation analysis between expression of MIR4435-2HG and EPCAM in cohort2. B Correlation analysis between expression of MIR4435-2HG and CD44 in cohort2. C Correlation analysis between expression of MIR4435-2HG and CD133 in cohort2. D Correlation analysis between expression of MIR4435-2HG and CD24 in TCGA-LIHC cohort. E Correlation analysis between expression of MIR4435-2HG and CD44 in TCGA-LIHC cohort. Fig. S3. MIR4435-2HG contributes to the sphere formation ability of HCC cells. A Representative pictures of tumor spheroids formed with MIR4435-2HG overexpression or control. B Representative pictures of tumor spheroids formed with MIR4435-2HG interference or control. Scale bar, 50 μm. Fig. S4. Co-IP was conducted to detect the interaction between NOP58 and IGF2BP1 in Huh7. Fig. S5. MIR4435-2HG dose not affect the expression of IGF2BP1 in HCC cells. A Overexpression of MIR4435-2HG did not affect mRNA levels of IGF2BP1 in HCC cells. B Overexpression of MIR4435-2HG did not affect protein levels of IGF2BP1 in HC [file 11658_2023_493_MOESM1_ESM.docx]

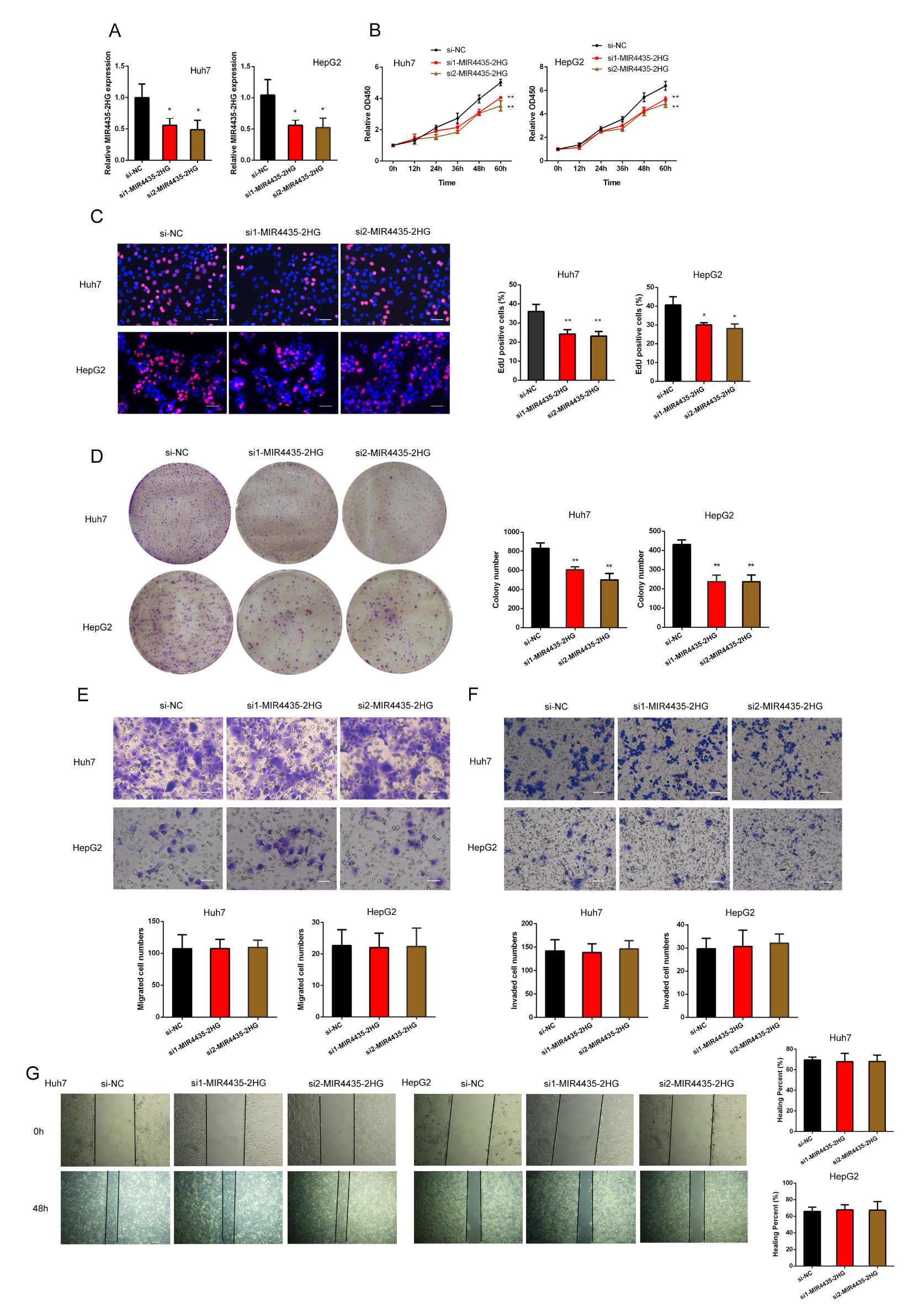


Fig. S1. Knockdown of MIR4435-2HG decreases the progression of HCC cells.

**A** MIR4435-2HG expression in HCC cells with siRNA transfection. **B** Cellular proliferation of HCC cells with MIR4435-2HG interference or control was detected by CCK8 assay. **C** Cellular proliferation of HCC cells with MIR4435-2HG interference or control was detected by EdU assay. **D** Colony formation ability of HCC cells with MIR4435-2HG interference or control was detected. The data are shown as mean ± SD. **E** Migration abilities of HCC cells with MIR4435-2HG interference or control was detected by transwell migration assay. **F** Invasion abilities of HCC cells with MIR4435-2HG interference or control was detected by transwell invasion assay. **G** Migration abilities of HCC cells with MIR4435-2HG interference or control was detected by wound healing assay. ^*^*P* < 0.05, ^**^*P* < 0.01. Scale bar, 50 μm.


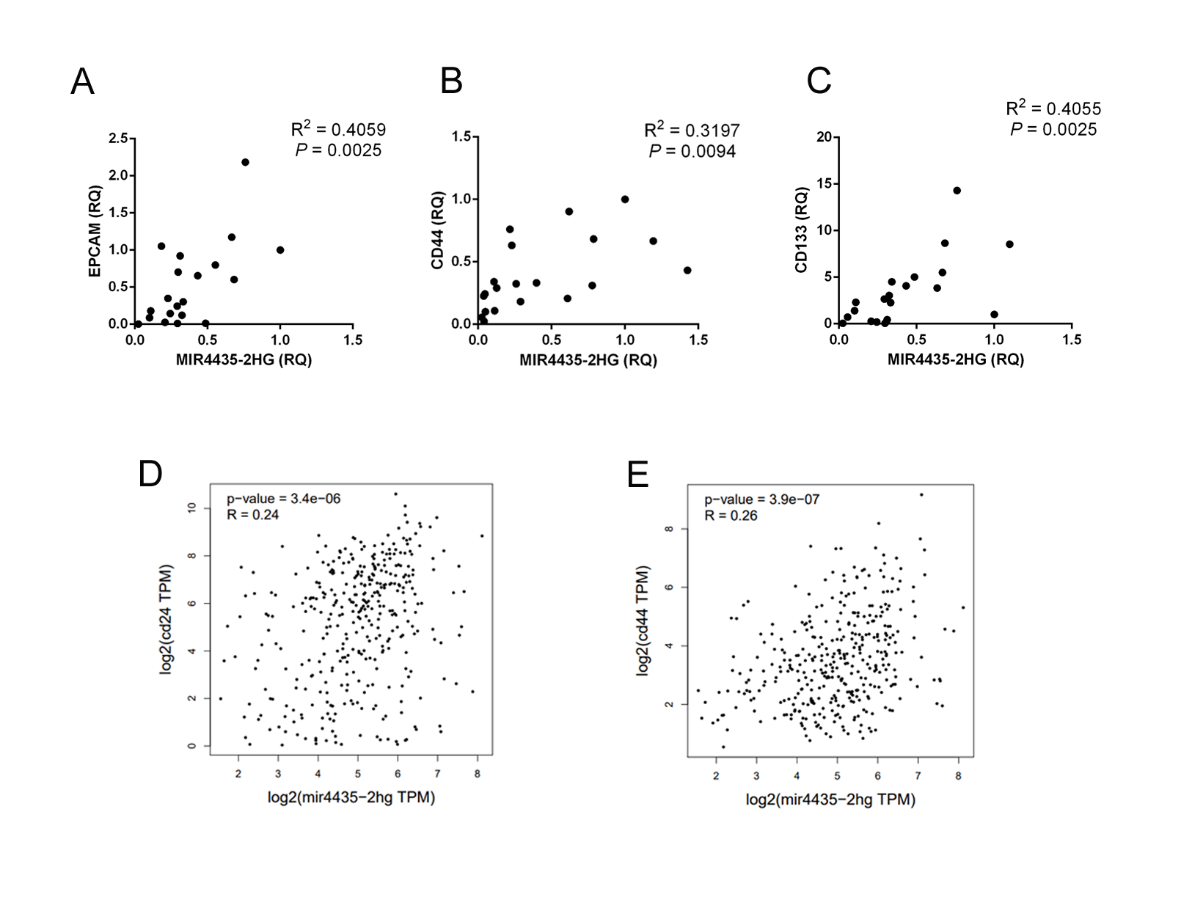


Fig. S2. The relationship between the expression of MIR4435-2HG and stem cell markers.

**A** Correlation analysis between expression of MIR4435-2HG and EPCAM in cohort2. **B** Correlation analysis between expression of MIR4435-2HG and CD44 in cohort2. **C** Correlation analysis between expression of MIR4435-2HG and CD133 in cohort2. **D** Correlation analysis between expression of MIR4435-2HG and CD24 in TCGA-LIHC cohort. **E** Correlation analysis between expression of MIR4435-2HG and CD44 in TCGA-LIHC cohort.


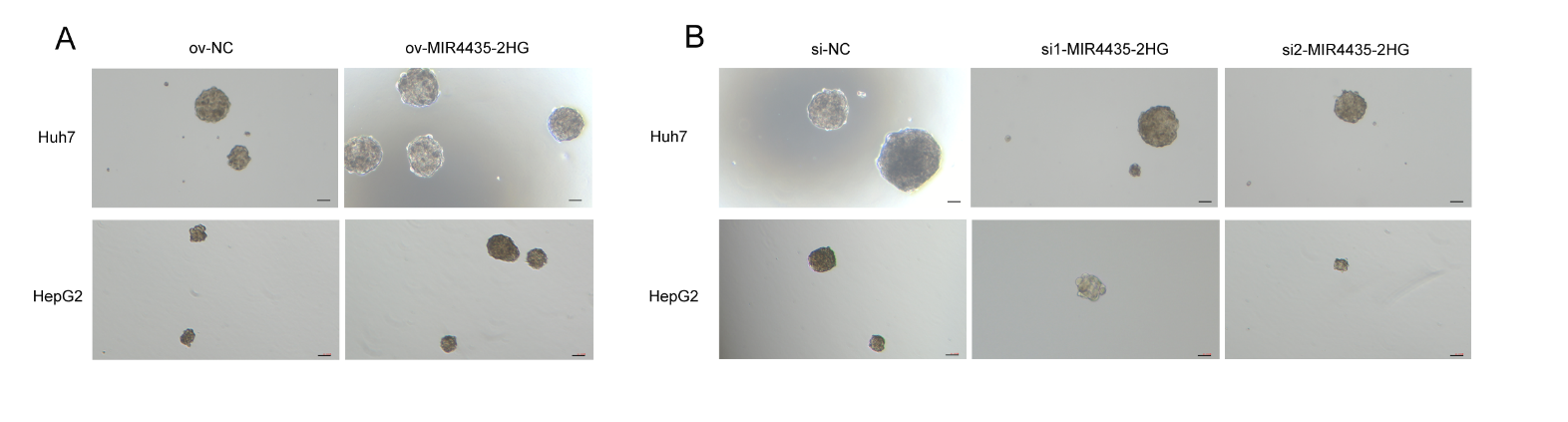


Fig. S3. MIR4435-2HG contributes to the sphere formation ability of HCC cells.

**A** Representative pictures of tumor spheroids formed with MIR4435-2HG overexpression or control. **B** Representative pictures of tumor spheroids formed with MIR4435-2HG interference or control. Scale bar, 50 μm.


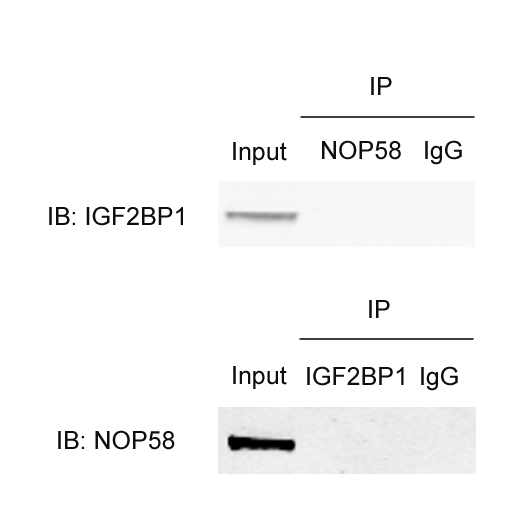


Fig. S4. Co-IP was conducted to detect the interaction between NOP58 and IGF2BP1 in Huh7.


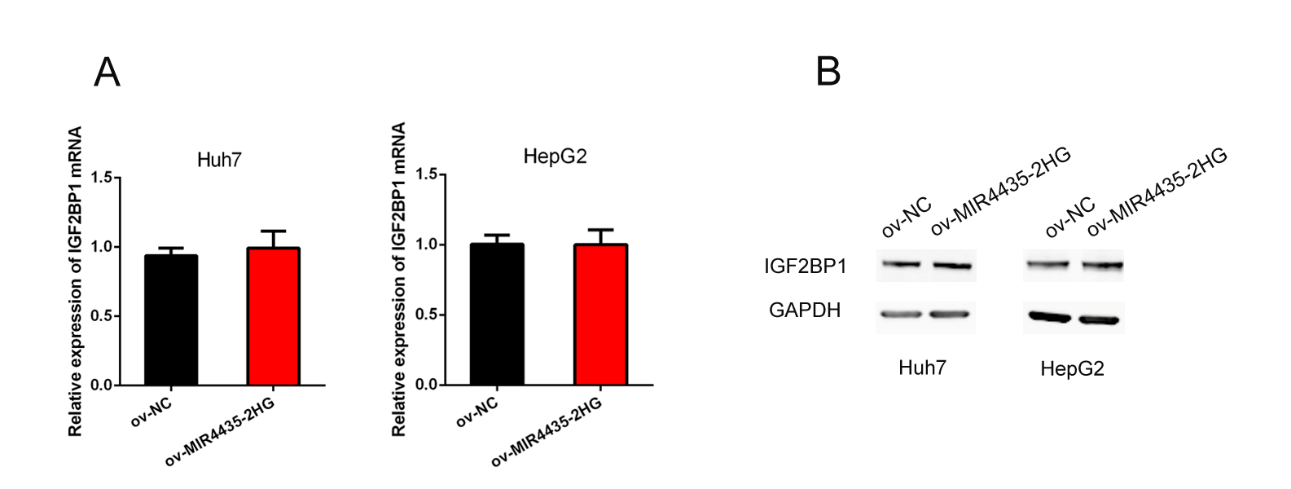


Fig. S5. MIR4435-2HG dose not affect the expression of IGF2BP1 in HCC cells.

**A** Overexpression of MIR4435-2HG did not affect mRNA levels of IGF2BP1 in HCC cells. **B** Overexpression of MIR4435-2HG did not affect protein levels of IGF2BP1 in HCC cells.


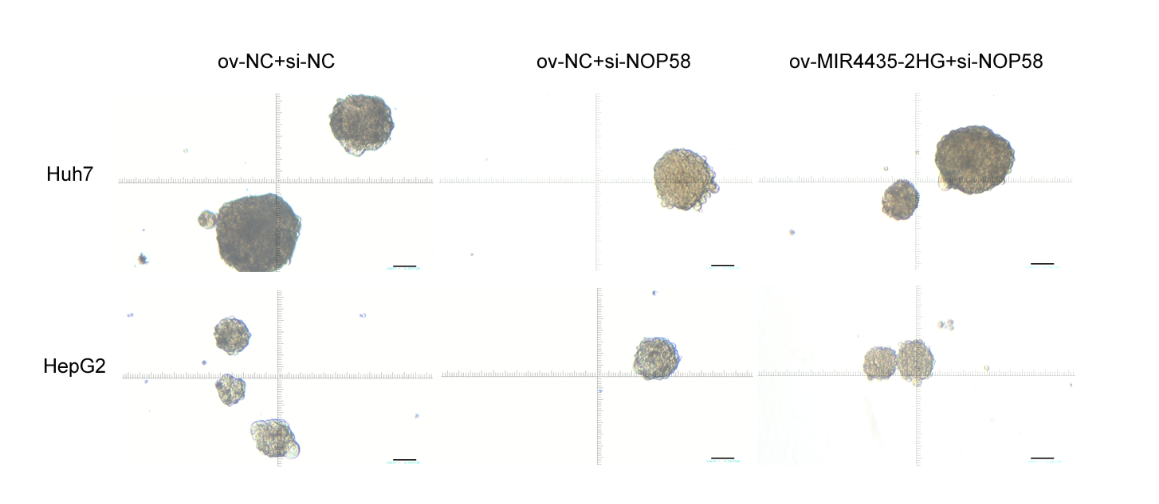


Fig. S6. Representative pictures of tumor spheroids formed with different treatment. Scale bar, 50 μm.


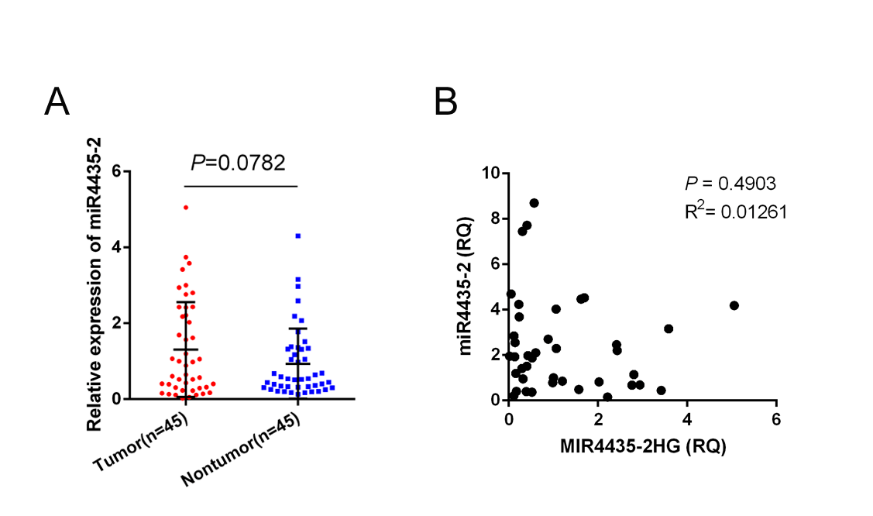


Fig. S7. The role of mir4435-2 in HCC.

A Relative expression of mir4435-2 in HCC and pair nontumor tissues. B The relationship between expression of mir4435-2 and MIR4435-2HG in HCC.


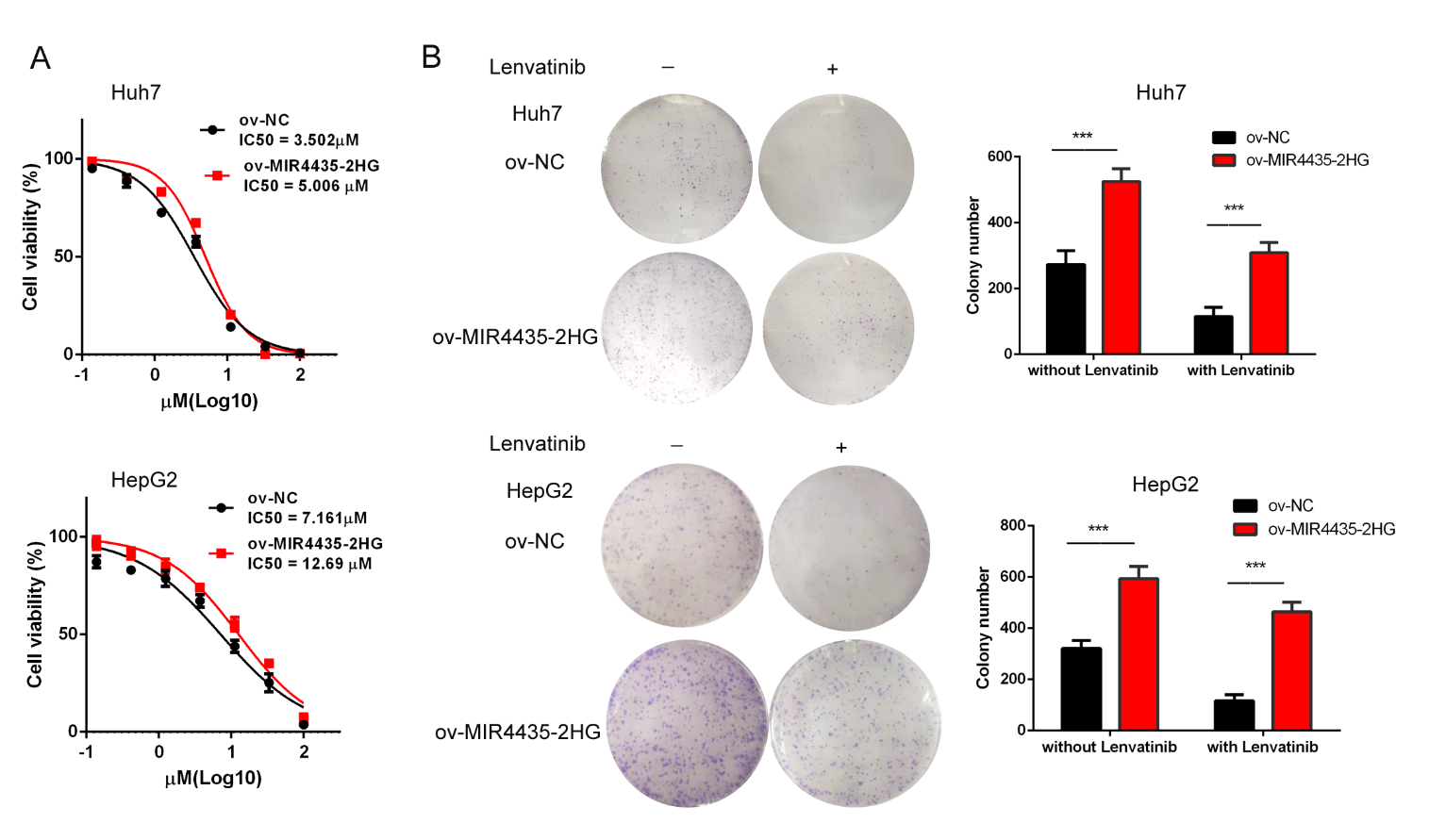


Fig. S8. The role of MIR4435-2HG in lenvatinib resistance.

A The IC50 value of Lenvatinib was measured in MIR4435-2HG overexpression and control HCC cells. B Colony formation assays were used to detect the sensitivity of HCC cells to Lenvatinib. ^***^*P* < 0.001.


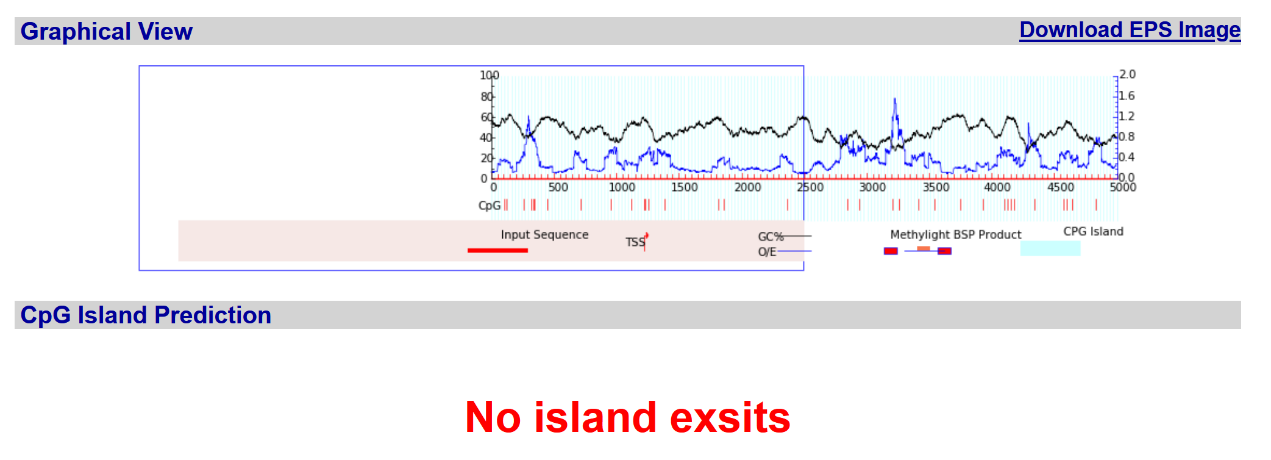


Fig. S9. The predicted DNA methylation modification in the promoter region of MIR4435-2HG by Methprimer.


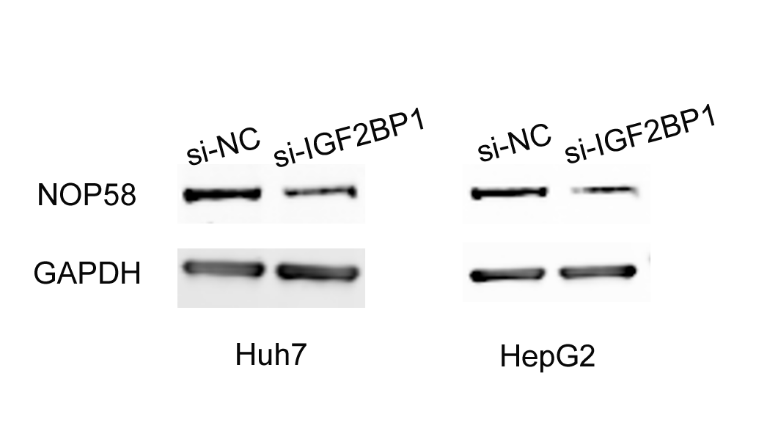


Fig. S10. Knockdown of IGF2BP1 decreased the expression of NOP58 in HCC cells.


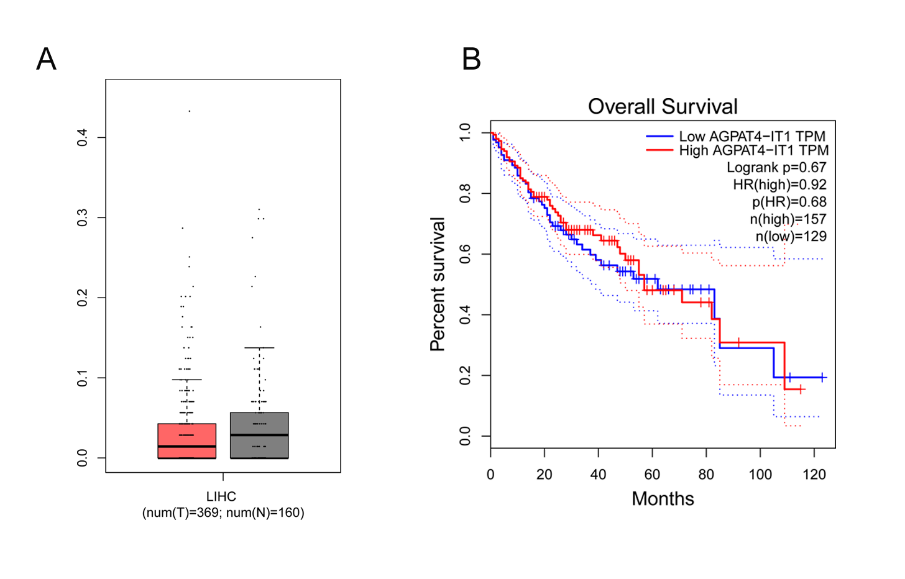


Fig. S11. Expression of AGPAT4-IT1 in HCC patients from the TCGA-LIHC cohort.

A Expression of AGPAT4-IT1 in HCC and nontumor tissues in TCGA-LIHC cohort. B Keplan-Meier’s survival curves of OS in TCGA-LIHC cohort.
