# Supplementary material for: N6-methyladenosine-modified oncofetal lncRNA MIR4435-2HG contributed to stemness features of hepatocellular carcinoma cells by regulating rRNA 2′-O methylation
Source: Cell Mol Biol Lett. 2023 Oct 27;28:89. doi: 10.1186/s11658-023-00493-2 (PMC10612268; doi:10.1186/s11658-023-00493-2)
Supplement: Supplementary file 2 — Additional file 2: Table S1. Sequences of primers for qPCR and RTL-P and siRNA used in this study. Table S2. Mass spectrometry analysis of the gel. [file 11658_2023_493_MOESM2_ESM.docx]

Table S1. Sequences of primers for qPCR and RTL-P and siRNA used in this study

| Name | Sequence |
| --- | --- |
| MIR4435-2HG-forward | ACTGGTGAAGGCATCGTG |
| MIR4435-2HG-reverse | GTCCTGGTGAGTGTTGTGGT |
| NOP58-forward | AGCACGAGTCATCTGGTTCC |
| NOP58-reverse | ACCCAGTCCCCTCATTCTCA |
| IGF2BP1-forward | GCGGCCAGTTCTTGGTCAA |
| IGF2BP1- reverse | TTGGGCACCGAATGTTCAATC |
| CD24-forward | CTCCTACCCACGCAGATTTATTC |
| CD24- reverse | AGAGTGAGACCACGAAGAGAC |
| CD44-forward | CTGCCGCTTTGCAGGTGTA |
| CD44- reverse | CATTGTGGGCAAGGTGCTATT |
| CD133-forward | AGTCGGAAACTGGCAGATAGC |
| CD133- reverse | GGTAGTGTTGTACTGGGCCAAT |
| EPCAM-forward | AATCGTCAATGCCAGTGTACTT |
| EPCAM - reverse | TCTCATCGCAGTCAGGATCATAA |
| NOTCH-1-forward | GAGGCGTGGCAGACTATGC |
| NOTCH-1- reverse | CTTGTACTCCGTCAGCGTGA |
| U6-forward | ATTGGAACGATACAGAGAAGATT |
| U6-reverse | GGAACGCTTCACGAATTTG |
| MIR4435-2-forward | GCAAATGGCCAGAGCTCACA |
| β-actin-forward | GGGAAATCGTGCGTGACATTAAG |
| β-actin-reverse | TGTGTTGGCGTACAGGTCTTTG |
| 18S G1328-forward | TCTTTCTCGATTCCGTGGGTG |
| 18S G1328-reverse | CATGCCAGAGTCTCGTTCGT |
| 18S G1490-forward | TTCAGCCACCCGAGATTGAG |
| 18S G1490-reverse | CGCTGAGCCAGTCAGTGTAG |
| 18S C1703-forward | GCGTTGATTAAGTCCCTGCC |
| 18S C1703-reverse | GGGCCTCACTAAACCATCCA |
| 28S U4197-forward | TCAAACGGTAACGCAGGTGT |
| 28S U4197-reverse | GATCAAGCGAGCTTTTGCCC |
| 28S C4506-forward | GCGTTGGATTGTTCACCCAC |
| 28S C4506-reverse | ACCATGGCAACAACACATCA |
| si1-MIR4435-2HG-sense | GCAGAAGACAAAGCCGAAUGC |
| si1-MIR4435-2HG-anti-sense | GCAUUCGGCUUUGUCUUCUGC |
| si2-MIR4435-2HG-sense | GGUCUGGUCGGUUUCCCAUUU |
| si2-MIR4435-2HG-anti-sense | AAAUGGGAAACCGACCAGACC |
| si-METTL3-sense | GCAAGUAUGUUCACUAUGATT |
| si-METTL3-anti-sense | UCAUAGUGAACAUACUUGCAG |
| si-NOP58-sense | GCGAUGUAGAGAAUGGUAUTT |
| si-NOP58-anti-sense | AUACCAUUCUCUACAUCGCTT |
| si-IGF2BP1-sense | CCGGGAGCAGACCAGGCAA |
| si-IGF2BP1 -anti-sense | UGAAUGGCCACCAGUUGGA |
| si-NC-sense | UUCUCCGAACGUGUCACGUTT |
| si-NC-anti-sense | ACGUGACACGUUCGGAGAATT |

Table S2. Mass spectrometry analysis of the gel

| Gene Symbol | Coverage | Unique Peptides |
| --- | --- | --- |
| IGF2BP1 | 43.5% | 22 |
| NOP58 | 33.67% | 16 |
