# Supplementary material for: N6-methyladenosine-modified oncofetal lncRNA MIR4435-2HG contributed to stemness features of hepatocellular carcinoma cells by regulating rRNA 2′-O methylation
Source: Cell Mol Biol Lett. 2023 Oct 27;28:89. doi: 10.1186/s11658-023-00493-2 (PMC10612268; doi:10.1186/s11658-023-00493-2)
Supplement: Supplementary file 3 — Additional file 3: Raw images from gels or western blots. [file 11658_2023_493_MOESM3_ESM.docx]

Western blots for Figure 4B

IGF2BP1


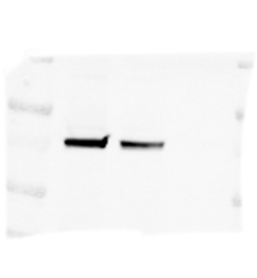

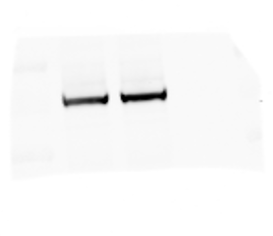

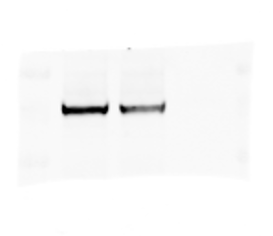


100kDa

70kDa

55kDa

100kDa

70kDa

55kDa

55kDa

70kDa

100kDa

NOP58


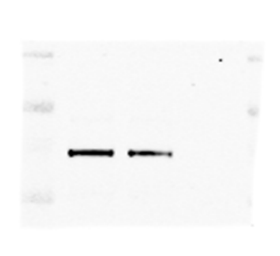

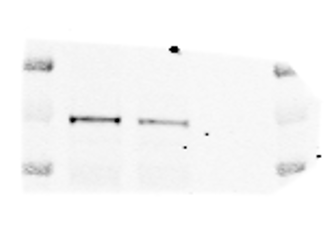

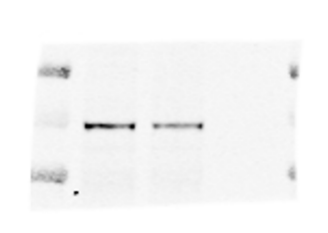


55kDa

55kDa

55kDa

100kDa

100kDa

100kDa

70kDa

70kDa

70kDa

Western blot for Figure 4E

IGF2BP1


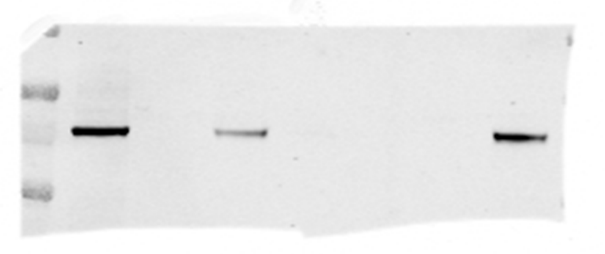

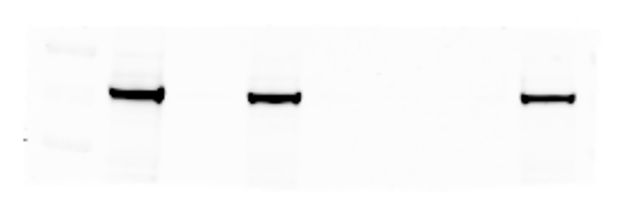


55kDa

70kDa

100kDa

100kDa

70kDa

55kDa


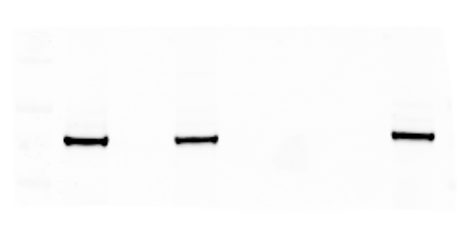


55kDa

70kDa

100kDa

NOP58


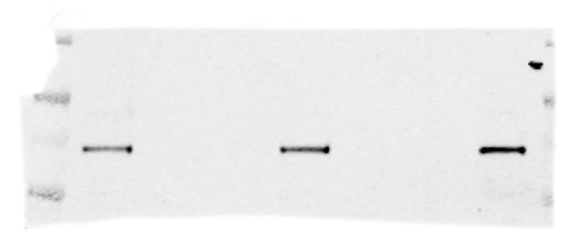

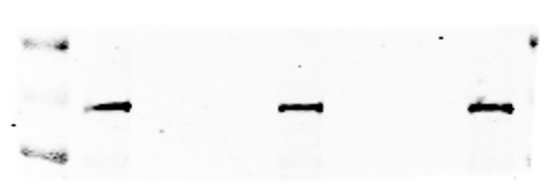


55kDa

70kDa

100kDa

70kDa

100kDa

55kDa


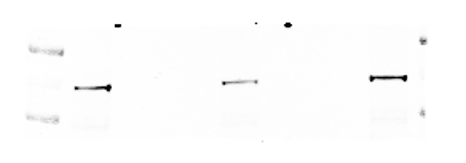


100kDa

70kDa

55kDa

Western blots for Figure 5A

IGF2BP1 in Huh7


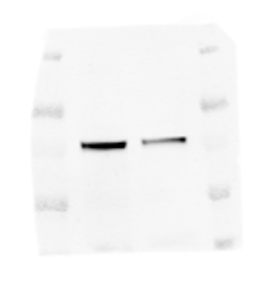

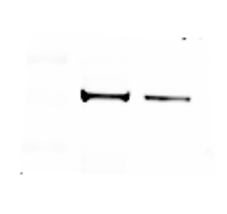

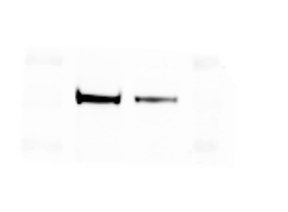


55kDa

70kDa

100kDa

55kDa

70kDa

100kDa

55kDa

70kDa

100kDa

IGF2BP1 in HepG2


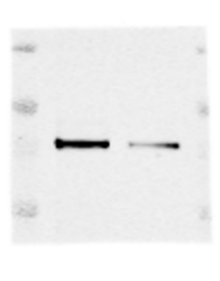

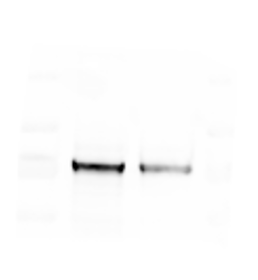

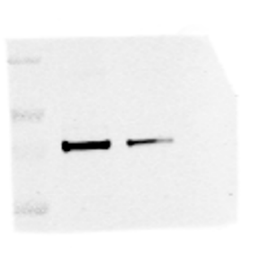


55kDa

70kDa

100kDa

55kDa

70kDa

100kDa

55kDa

70kDa

100kDa

GAPDH in Huh7


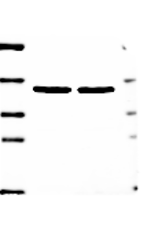

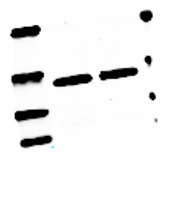

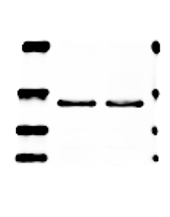


55kDa

40kDa

35kDa

35kDa

55kDa

40kDa

35kDa

40kDa

55kDa

GAPDH in HepG2


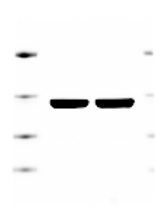

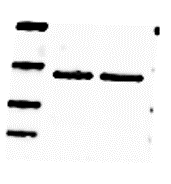

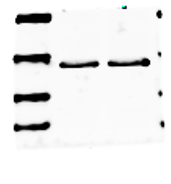


55kDa

40kDa

35kDa

55kDa

35kDa

40kDa

35kDa

40kDa

55kDa

Western blots for Figure 5E

METTL3 in Huh7


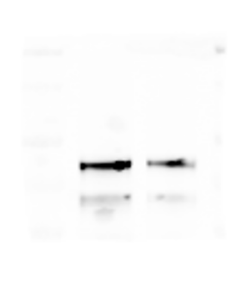

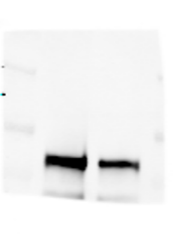

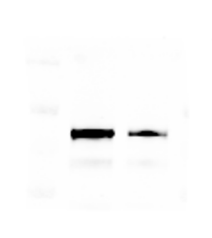


100kDa

70kDa

130kDa

55kDa

100kDa

70kDa

100kDa

55kDa

70kDa

METTL3 in HepG2


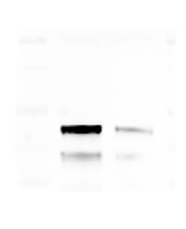

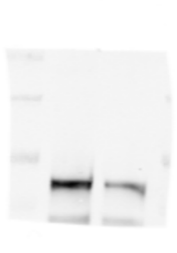

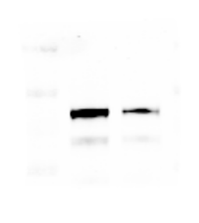


55kDa

70kDa

100kDa

130kDa

130kDa

70kDa

100kDa

100kDa

70kDa

55kDa

GAPDH in Huh7


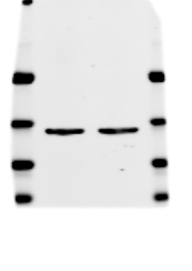

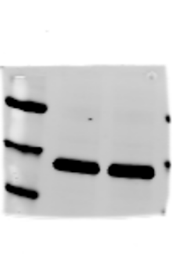

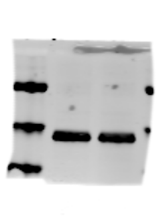


40kDa

40kDa

35kDa

35kDa

55kDa

55kDa

35kDa

40kDa

55kDa

GAPDH in HepG2


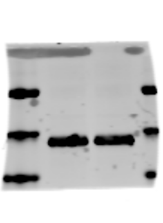

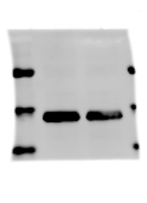

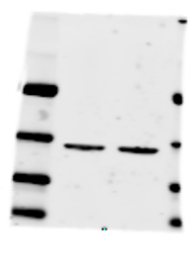


35kDa

35kDa

35kDa

55kDa

55kDa

55kDa

40kDa

40kDa

40kDa

Western blots for Figure 6B

NOP58 in Huh7


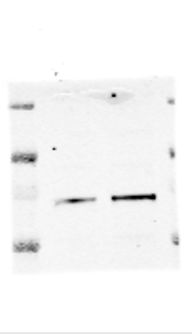

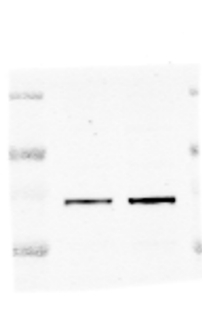

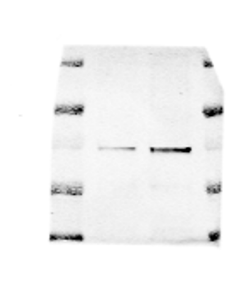


55kDa

55kDa

100kDa

100kDa

70kDa

70kDa

55kDa

100kDa

70kDa

GAPDH in Huh7


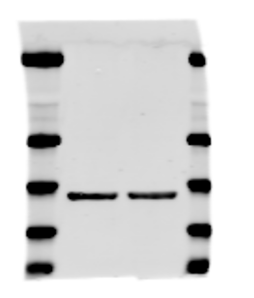

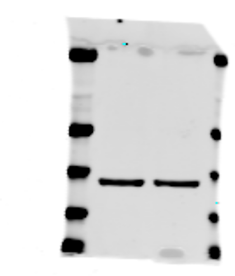

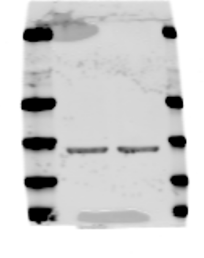


35kDa

35kDa

35kDa

40kDa

40kDa

40kDa

55kDa

55kDa

55kDa

NOP58 in HepG2


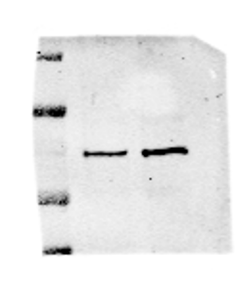

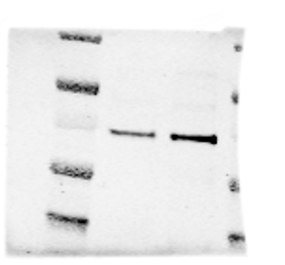

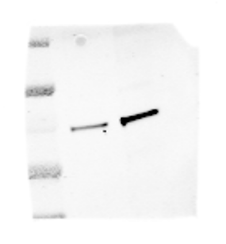


55kDa

55kDa

55kDa

70kDa

70kDa

70kDa

100kDa

100kDa

100kDa

GAPDH in HepG2


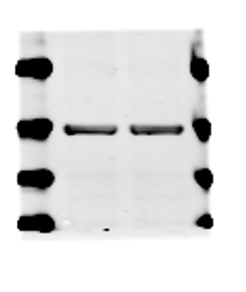

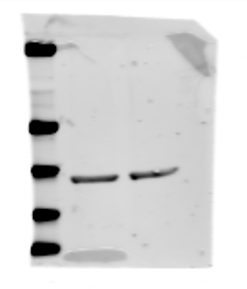

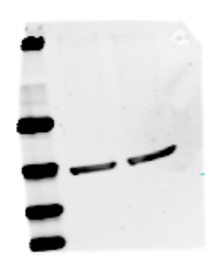


55kDa

55kDa

55kDa

40kDa

40kDa

40kDa

35kDa

35kDa

35kDa

Western blots for Figure 6C

NOP58 in Huh7 with ov-NC


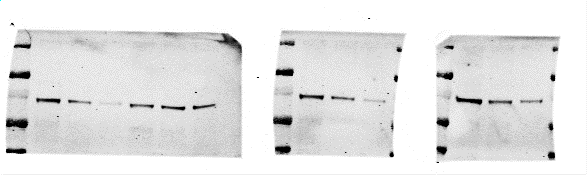

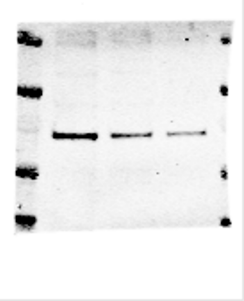

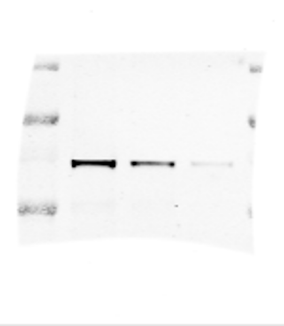


55kDa

55kDa

130kDa

70kDa

70kDa

130kDa

130kDa

70kDa

55kDa

GAPDH in Huh7 with ov-NC


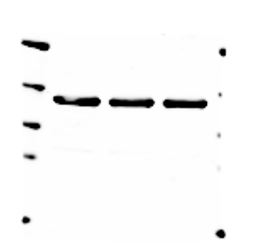

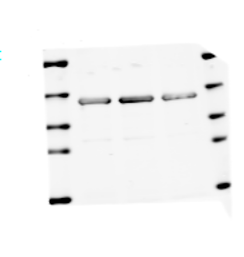

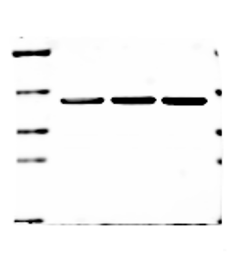


35kDa

35kDa

35kDa

40kDa

40kDa

40kDa

55kDa

55kDa

55kDa

NOP58 in Huh7 with ov-MIR4435-2HG






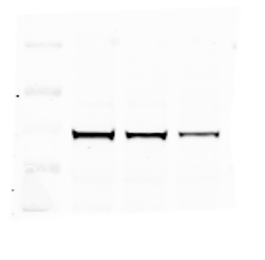


100kDa

100kDa

100kDa

70kDa

70kDa

70kDa

55kDa

55kDa

55kDa

GAPDH in Huh7 with ov-MIR4435-2HG


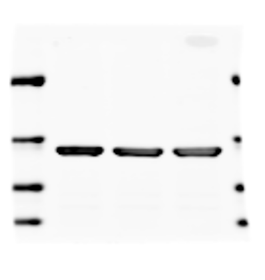

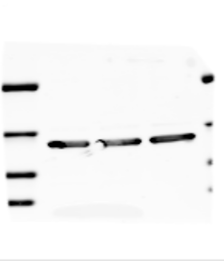

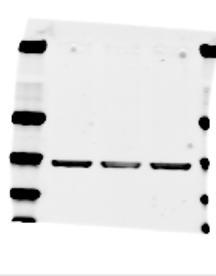


35kDa

35kDa

35kDa

40kDa

40kDa

40kDa

55kDa

55kDa

55kDa

NOP58 in HepG2 with ov-NC









55kDa

55kDa

55kDa

70kDa

70kDa

70kDa

100kDa

100kDa

100kDa

GAPDH in HepG2 with ov-NC


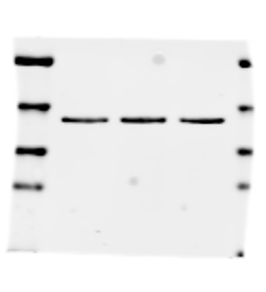


55kDa


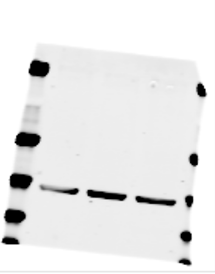

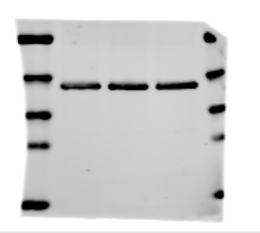

NOP58 in HepG2 with ov-MIR4435-2HG

55kDa

55kDa

40kDa

40kDa

40kDa

35kDa

35kDa

35kDa


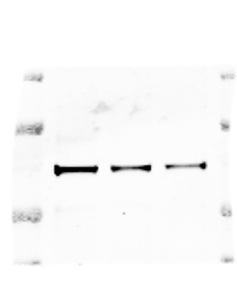

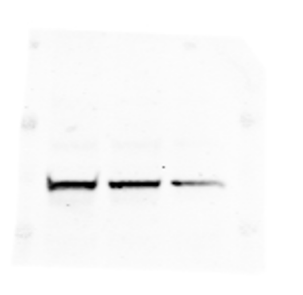

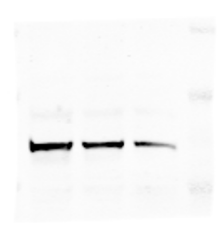


55kDa

55kDa

55kDa

70kDa

70kDa

70kDa

100kDa

100kDa

100kDa

GAPDH in HepG2 with ov-MIR4435-2HG


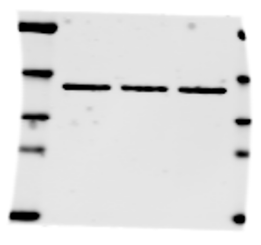

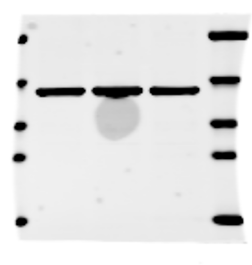

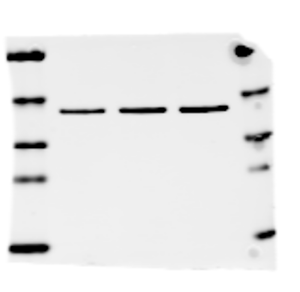


35kDa

35kDa

35kDa

40kDa

40kDa

40kDa

55kDa

55kDa

55kDa

Western blots for Figure 6D

NOP58 in Huh7


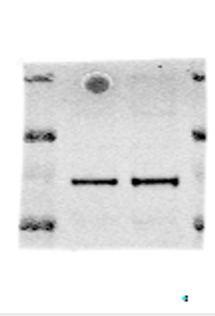

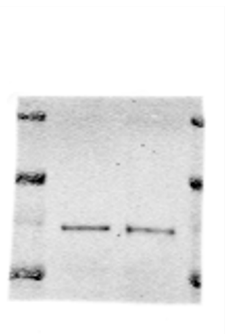

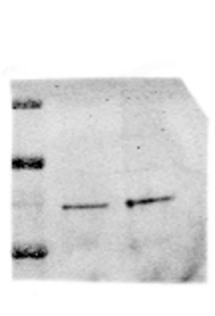


100kDa

100kDa

100kDa

70kDa

70kDa

70kDa

55kDa

55kDa

55kDa

GAPDH in Huh7


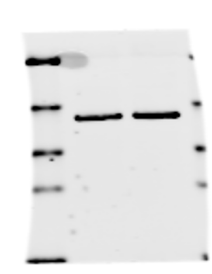

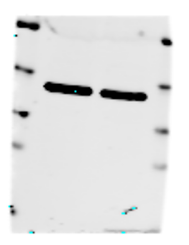

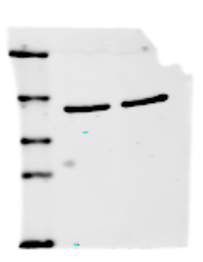


55kDa

55kDa

55kDa

40kDa

40kDa

40kDa

35kDa

35kDa

35kDa

NOP58 in HepG2


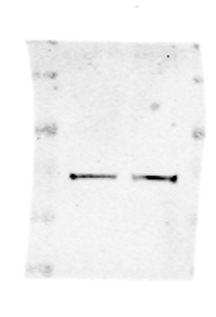

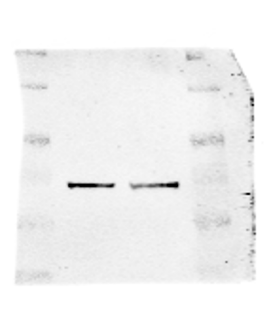

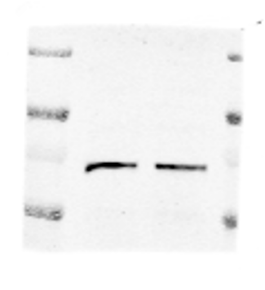


55kDa

55kDa

55kDa

70kDa

70kDa

70kDa

100kDa

100kDa

100kDa

GAPDH in HepG2


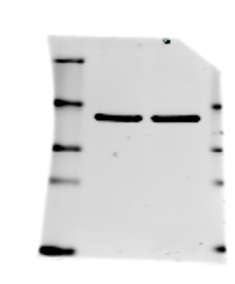

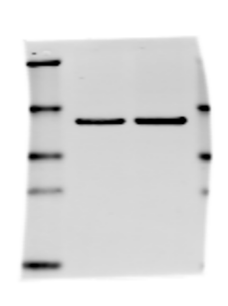

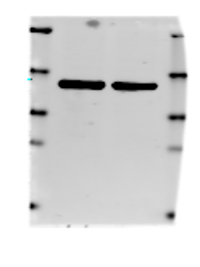


35kDa

35kDa

35kDa

40kDa

40kDa

40kDa

55kDa

55kDa

55kDa

Western blots for Figure 6G

NOP58 in Huh7


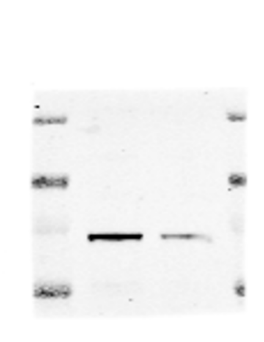

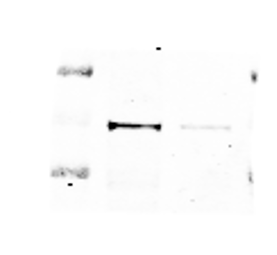

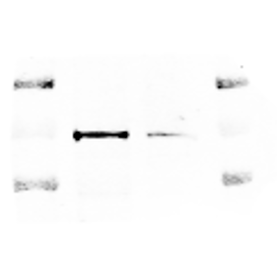


100kDa

100kDa

100kDa

70kDa

70kDa

70kDa

55kDa

55kDa

55kDa

NOP58 in HepG2


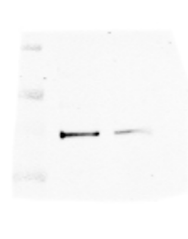

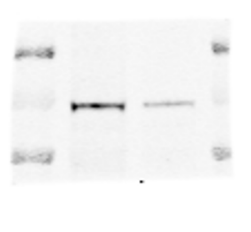

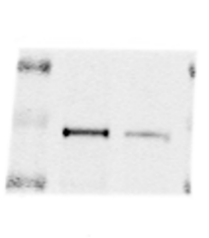


55kDa

55kDa

55kDa

70kDa

70kDa

70kDa

100kDa

100kDa

100kDa

GAPDH in Huh7


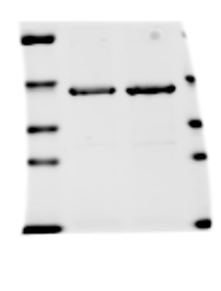

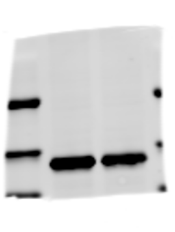

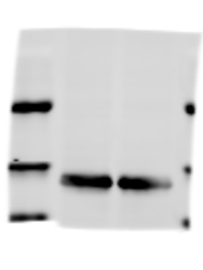


35kDa

35kDa

35kDa

40kDa

40kDa

40kDa

55kDa

55kDa

55kDa

GAPDH in HepG2


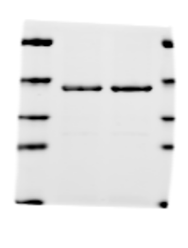

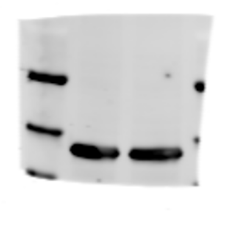

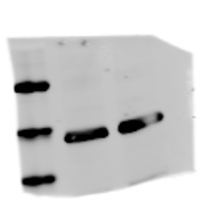


55kDa

55kDa

55kDa

40kDa

40kDa

40kDa

35kDa

35kDa

35kDa

Western blots for Figure 7F

IGF1R in Huh7


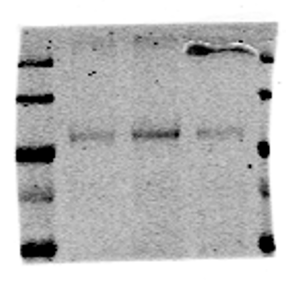

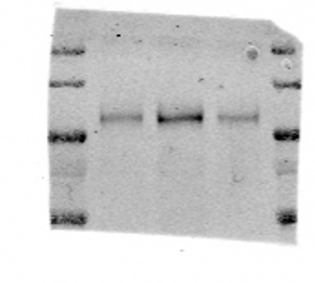

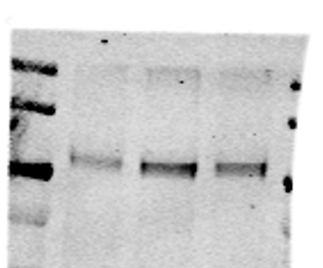


180kDa

180kDa

180kDa

130kDa

130kDa

130kDa

100kDa

100kDa

100kDa

MYC in Huh7


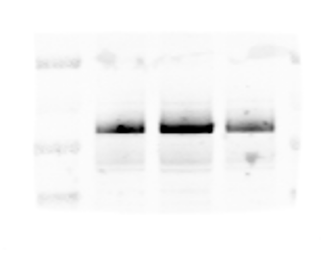


100kDa

100kDa

100kDa

70kDa

70kDa

70kDa

55kDa

55kDa

55kDa

GAPDH in Huh7

55kDa

55kDa

55kDa

35kDa

35kDa

35kDa

40kDa

40kDa

40kDa

IGF1R in HepG2

180kDa

180kDa

180kDa

130kDa

130kDa

130kDa

100kDa

100kDa

100kDa

MYC in HepG2

40kDa

100kDa

100kDa

70kDa

70kDa

70kDa

55kDa

55kDa

55kDa

GAPDH in HepG2

35kDa

35kDa

35kDa

55kDa

55kDa

55kDa

40kDa

40kDa

40kDa

Western blots for Figure S4B

IB: IGF2BP1

100kDa

100kDa

100kDa

70kDa

70kDa

70kDa

55kDa

55kDa

55kDa

IB: NOP58

55kDa

55kDa

55kDa

70kDa

70kDa

70kDa

100kDa

100kDa

100kDa

Western blots for Figure S5B

IGF2BP1 in Huh7

100kDa

100kDa

100kDa

70kDa

70kDa

55kDa

70kDa

55kDa

55kDa

GAPDH in Huh7

35kDa

35kDa

35kDa

40kDa

40kDa

40kDa

55kDa

55kDa

55kDa

IGF2BP1 in HepG2

55kDa

55kDa

55kDa

70kDa

70kDa

70kDa

100kDa

100kDa

100kDa

GAPDH in HepG2

55kDa

55kDa

55kDa

40kDa

40kDa

40kDa

35kDa

35kDa

35kDa

Western blots for Figure S10

NOP58 in Huh7

100kDa

100kDa

70kDa

70kDa

70kDa

55kDa

55kDa

55kDa

100kDa

GAPDH in Huh7

40kDa

40kDa

40kDa

55kDa

55kDa

55kDa

35kDa

35kDa

35kDa

NOP58 in HepG2

55kDa

55kDa

55kDa

70kDa

70kDa

70kDa

100kDa

100kDa

100kDa

GAPDH in HepG2

35kDa

35kDa

35kDa

55kDa

55kDa

40kDa

40kDa

40kDa

55kDa

Gel for Figure 4A

100kDa

70kDa

55kDa
